# Extending the liaison psychiatry service in a large hospital in the UK: a before and after evaluation of the economic impact and patient care following ED attendances for self-harm

**DOI:** 10.1136/bmjopen-2017-016906

**Published:** 2017-08-21

**Authors:** Brent C Opmeer, William Hollingworth, Elsa M R Marques, Ruta Margelyte, David Gunnell

**Affiliations:** 1 The National Institute for Health Research Collaboration for Leadership in Applied Health Research and Care (CLAHRC) West at University Hospitals Bristol, Bristol, UK; 2 Clinical Research Unit, Academic Medical Centre, Amsterdam, The Netherlands; 3 School of Social and Community Medicine, University of Bristol, Bristol, UK; 4 School of Clinical Sciences, University of Bristol, Bristol, UK

**Keywords:** psychosocial assesement, service evaluation, liaison psychiatry

## Abstract

**Objectives:**

To evaluate the impact of an expansion of liaison psychiatry services (LPS) on patient management, outcomes and treatment costs for emergency department (ED) attendances for self-harm.

**Design:**

Retrospective before and after cohort study using routinely collected Self-Harm Surveillance Register data.

**Setting:**

A large hospital in South West England.

**Subjects:**

Patients attending the ED for self-harm.

**Interventions:**

Extension of the LPS’ working hours from 9:00 to 17:00, Monday to Friday to 8:00 to 22:00, 7 days a week, following a £250 000 annual investment

**Main outcome measures:**

Number and characteristics of ED attendances for self-harm. The before and after cohorts were compared in terms of key process measures, including proportion of patients receiving a psychosocial assessment, average length of hospital stay, waiting times for assessment, proportion of patients who self-discharged without an assessment, levels of repeat self-harm attendances and mean cost per patient attendance.

**Results:**

298 patients attended ED for self-harm on 373 occasions between January and March 2014, and 318 patients attended on 381 occasions between January and March 2015. The proportion of ED attendances where patients received a psychosocial assessment increased from 57% to 68% (p=0.003), median waiting time decreased by 3 hours and 14 min (p=0.017), and the proportion of episodes where patients self-discharged without a psychosocial assessment decreased from 20% to 13% (p=0.022). The mean cost per patient attendance was marginally lower after the intervention (−£84; 95% CI −£254 to £77).

**Conclusions:**

The extended LPS seems to have had a favourable effect on the management and outcomes of self-harm patients. The cost of extending the LPS’ working hours might be partially offset by more efficient assessment and discharge. The impact of the extended LPS on the care of hospitalised patients with mental health problems other than self-harm requires further evaluation.

**Strengths and limitations of this study**

- There was a major step-change in care provision (increase in service availability from 40 to 98 hours per week), providing a good opportunity to evaluate the impact of changed service provision.
- Detailed and relatively complete individual level patient data were available from a bespoke self-harm register, facilitating estimation of resource costs for self-harm patients.

- Analysis does not assess the wider impact of the extended LPS on postdischarge service provision and on patients with other mental health conditions seen by the LPS.
- Analysis does not include a control site for wider control of economic, social and political trends in mental health and service provision.
- The sample size was relatively small and we lacked the power to detect important impacts on hospital costs.

## Introduction

There are an estimated 200 000 emergency department (ED) attendances for self-harm in England and Wales every year^1^; approximately half of these result in admission to a hospital ward.^2^ Self-harm is often repeated, with more than 15% of individuals who attend a hospital with self-harm reattending within a year.^3^ A history of self-harm is the strongest risk factor for suicide across a range of psychiatric disorders.^4^ Repeated self-harm further increases suicide risk.^5^ Providing effective, evidence-based clinical care for this high-risk patient population is a key means of reducing their risk of subsequent self-harm and suicide.

UK clinical guidelines suggest that all patients should be offered a psychosocial assessment after self-harm. This should include an evaluation of the factors leading to self-harm and suicidal intent together with a full mental health and social needs assessment.^6^ However in many UK hospitals, more than half of patients are discharged from the ED without an assessment.^2^ Patients who leave the ED without a psychosocial assessment are less likely to be offered follow-up.^7^ There is also evidence that psychosocial assessments reduce risk of repeat self-harm.^8 9^

Liaison psychiatry services (LPS) have been introduced in hospitals to provide assessment and care for patients presenting to the ED with mental health problems and to support people with physical health problems who also have, or develop, mental health problems such as delirium while they are in hospital. Despite the existing guidelines on the management of self-harm,^6^ there are significant variations in service models in terms of staffing, coverage or management.^2 10^ Most people who have self-harmed seek help at times when only an emergency mental health service is available. One response is to invest in extended LPS operating hours, but the benefits of such investment have not been well established. An exception is the economic evaluation of expanded LPS service at a large acute hospital in Birmingham which found that additional investment in the services generated incremental benefits in terms of reduced bed use with overall benefit to cost ratio of more than 4:1.^11^

In 2014, a local clinical commissioning group invested approximately £250 000 per annum in an extended LPS at a large teaching hospital with a consultant-led 24 hours ED. This was used to increase the working hours of the LPS from Monday to Friday 09:00–17:00 (ie, 40 hours per week) to 7 days a week 08:00–22:00 (98 hours per week). To achieve this increase in service provision, four additional full-time liaison nurses were employed. The aim was to increase the proportion of patients attending the ED after self-harm who receive a psychosocial assessment and reduce admissions to acute hospital beds to await LPS assessment. It was anticipated that these changes might also lead to better patient outcomes such as reduced repeat self-harm and suicide as a result of increases in the proportion of patients receiving appropriate follow-up care.

Our primary objective was to assess the impact of the extended LPS on process measures and indicators of patient outcomes and costs following self-harm. We assessed resource use and costs associated with the management of patients attending the ED following self-harm. Our findings will enable commissioners to explore whether extra investment in LPS can improve patient management and outcomes of self-harm and result in cost savings.

## Methods

### Study design

The change in LPS provision is a natural experiment^12^ as there has been a step-change in the availability of a service from a defined point in time (1September 2014). We compared the process, cost and outcomes of care for patients attending the ED following self-harm in a before and after study. We estimated National Health Service (NHS) secondary care costs, although we recognise that care provided by the LPS will have spillover effects on primary and community care services.

### Participants and data

We compared two patient cohorts. The first consisted of patients presenting to the ED following self-harm before the operating hours of LPS were extended (1 January–31 March 2014). The second consisted of patients presenting after the extended LPS was fully operational (1 January–31 March 2015). Our focus was on self-harm patients because these are the group of psychiatric patients most likely to be admitted to a hospital bed, and they were the focus of the new funding. However, it is important to note that self-harm patients comprise only 40% of the LPS workload, therefore the extended service will have an impact on a wider group of patients.

Patients were identified from the local Self-Harm Surveillance Register (SHSR). The SHSR was established in 2010 and records clinical and sociodemographic details of all hospital-presentations for self-harm at the hospital. Using the SHSR, we identified the index self-harm ED attendances in each period (ie, the first time a patient attended between 1 January and 31 March) and any repeat self-harm ED attendance within 90 days of the index episode. The follow-up period was defined on the basis that a high proportion of all patients who repeat self-harm within 12 months do so within 90 days of the index event.^13^

We selected time periods which are not adjacent to the service change date (September 2014) to avoid the period when the service might have been ‘ramping up’ or ‘bedding in’. We selected the same three calendar months for the before and after periods to avoid bias due to seasonal trends in mental health and self-harm.

Each episode of self-harm was characterised in terms of patient age, sex, employment status, marital status, method of self-harm, previous self-harm or inpatient psychiatric care and a matrix risk assessment. The matrix is a locally developed tool for use by ED staff to determine the urgency with which a patient should be referred for psychosocial assessment. Patients are categorised into three groups (red, amber or green) depending on the degree of urgency. These levels guide clinical staff in deciding whether a patient should be referred for an immediate psychosocial assessment.

We assessed the following measures of care: whether a patient received a psychosocial assessment; the profession of the person carrying out the assessment; waiting times from attendance to assessment; proportion of patients self-discharging from the ED without assessment; proportion of episodes admitted to a hospital ward; referrals made to other agencies/health teams and length of hospital stay.

The patient outcome measures we evaluated were: the proportion of patients with repeat ED attendances for self-harm; number of repeat self-harm ED attendances and time to repeated self-harm attendance. We also evaluated mean cost per self-harm ED episode and mean cost per patient (including repeat self-harm episodes within 90 days).

Data collection is approved by the Central Bristol Research Ethics Committee.

### Data quality/missing data

Audits reveal SHSR case ascertainment is >95%. Data extracted from the SHSR were checked for inconsistencies, and where evident these were resolved.

The two main types of inconsistency that were identified were date errors (eg, dates of discharge preceded data of attendance) and variables reflecting composite questions, where the first variable was missing (eg, admitted to ward), but the second variable was completed (eg, date of admission to ward). In these evident cases, inconsistent or missing values were corrected with consistent values.

Multiple imputation (SAS V.9.4, PROC MI) was used, with 15 imputation rounds, to avoid exclusion of observations due to missing data in multivariable analyses (see below). Estimates of effects and standard errors from analyses based on each imputed dataset were subsequently pooled (SAS PROC MIANALYZE), reflecting the uncertainty due to the imputation of missing values.

### Statistical analyses

Analyses of patient characteristics were based on index attendances during the 3-month periods (January-March) in 2014 and 2015. Analyses of the impact on service delivery are based on all attendances (including repeat presentations) in each 3-month period (in 2014 and 2015). Analyses describing the impact on risk of repeat self-harm are based on index attendances and all subsequent attendances for repeat self-harm within 90 days associated with these index attendances (ie, including attendances up to June).

### Descriptive analyses

Characteristics of the study population in each 3-month period are reported descriptively. Continuous variables are summarised as medians, means and SD as appropriate, for categorical variables, the number and percentage of participants/attendances within each category are presented.

### Evaluation of impact

Differences in the process of care are examined for attendances before and after the extended LPS became operational. Proportions, means and medians for service outcome measures by year are reported with the absolute difference between the years and associated p values (two tailed χ^2^ or t-test). This includes the proportion of attendances that received a psychosocial assessment within or outside LPS service hours, the professional background of the assessor, the median times from ED arrival or medical assessment to psychosocial assessment, the proportion of attendances admitted to an observation ward, general ward or intensive therapy unit (ITU) and the median duration of hospital stay after admission. As the length of ITU care for patients admitted to the ITU was not documented, we assumed this duration to be 30% of the total stay, with a minimum of 1 day, for these patients. The proportion of patients that self-discharged and the number of episodes of repeated self-harm within 90 days are also considered as potential outcomes of the LPS activities.

Kaplan-Meier analyses were used to compare differences in time between ED arrival and psychosocial assessment before and after extended LPS.

Differences in time until repeat self-harm attendances within 90 days were also compared for 2014 and 2015 using Kaplan-Meier analyses and Cox proportional hazards regression to adjust for relevant factors (previous self-harm, age, sex and matrix risk) associated with reattendance rates. Time to repeat self-harm was compared before and after the extended LPS for all patients and for subgroups of patients with and without previous self-harm. We used SAS V.9.4 PROC PHREG, a proportional hazards regression analysis that allowed for multiple repeat self-harm episodes during the 90 days follow-up and used the robust ‘sandwich’ estimator to account for correlated observations within the same patient (ie, that some patients are more likely to repeat self-harm than others).^14^

### Economic analysis

Mean cost per attendance was estimated for patients presenting in the periods before and after the LPS was extended. This analysis was based on index presentation and all repeat attendances during the 90 days follow-up. All unit costs were estimated from the 2014/15 NHS reference costs.^15^ NHS reference costs for ED care are higher for patients subsequently admitted to a hospital bed (mean=£205.85) than for patients who are discharged from the ED (mean=£133.20). The NHS reference cost for an ED mental health liaison contact (£187) does not distinguish between those conducted by psychiatrists or nursing staff. We used this figure in our analysis, but the actual cost may be lower for assessments conducted by nurses. The cost of inpatient care depends on the ward type, the type of treatment required and the length of stay. Any reductions in hospital admissions due to the extended LPS may be due to fewer short stay admissions to observation units or other wards while waiting for psychosocial assessment. Therefore, we used the average unit cost (£405.50) for a non-elective short stay admission for ‘observation or counselling’ as a proxy for the per diem cost of observational unit or other ward care. We used the average daily cost (£1058.75) of adult medical critical care patients to estimate costs for ITU days. In view of the skewed distribution of healthcare costs, the SPSS V.23 bootstrap procedure was used to estimate 95% CIs for cost estimates, based on 1000 replications.

Robustness of the findings was assessed in the following univariate sensitivity analyses. We increased/decreased unit cost estimates for ward admissions as well as for LPS assessments by 25%. We differentiated between psychosocial assessment carried out by a liaison nurse or a psychiatrist by decreasing nurse costs and increasing psychiatrist costs by 25% each. Finally, we applied observational ward costs for all hospital days including ITU days, on the assumption that LPS may reduce hospital days but is very unlikely to have any effect on ITU days.

Data preparation, tables, figures and analyses are documented and performed using statistical software SPSS V.23, and SAS V.9.4.

## Results

Similar numbers of patients attended the ED following self-harm between January and March 2014 (n=298) and January and March 2015 (n=318) (table 1). Only around 20% of ED attendances in 2014 and 2015 occurred during the original LPS working hours (Monday–Friday, 09:00–17:00).

Overall, details in patient characteristics and patient care were accurately documented in the self-harm register. The number (%) of people with missing data are reported in tables 1 and 2. For key variables, for example, whether an assessment was performed, previous self-harm, time of assessment, time of discharge, outcome of ED attendance, completeness ranged from 0% (assessment performed/outcome of attendance) to 9.7% (previous self-harm).

**Table 1:** Number and characteristics of patients index episodes of ED SH attendances in 2014 and 2015 periods

	January– March 2014 (n=298)	January– March 2015 (n=318)
Attendances by hour of day n (%)		
Monday to Friday, 09:00–17:00	70 (21)	75 (24)
Other	228 (79)	242 (76)
Female n (%)	166 (57)	201 (63)
Age on years mean (SD)	34 (14)	35 (15)
Marital status n (%)		
Single	220 (74)	253 (80)
Married	30 (10)	32 (10)
Other	38 (13)	26 (8)
Unknown	10 (3)	7 (2)
Occupational status n (%)		
Employed	56 (19)	56 (18)
Unemployed	171 (57)	147 (46)
Other	55 (17)	80(25)
Unknown	14 (5)	35 (11)
Type of self-harm n (%)		
Self-poisoning	214 (72)	227 (71)
Self-injury	47 (16)	53 (17)
Both	21 (7.0)	23 (7.2)
Other/unknown	16 (5.3)	15 (4.7)
Previous self-harm n (%)		
Yes	215 (72)	364 (83)
No	54 (18)	46 (15)
Unknown	29 (9.7)	8 (2.5)
Previous inpatient psych treatment n (%)		
Yes	79 (75)	71 (73)
No	22 (21)	25 (26)
Unknown	4 (3.8)	1 (1.0)
Number of people presenting	298	318
Repeat episodes within January/February/March	75	63
Repeat episodes in April/May/June within 90 days from index episode	30	34
Total episodes	403	415
Number of self-harm episodes per patient (<90 days)		
1	250	265
2	36	34
3	5	11
4	2	2
>4	5	6
Max	19 (n=1)	9 (n=1)

ED SH, emergency department self-harm.

There are generally only minor differences in the characteristics of the self-harm patients in the two time periods. In 2015, a higher proportion of women attended following self-harm (63% vs 57%), and fewer patients were unemployed (46% vs 57%). Slightly more patients were known to have a history of self-harm (83% vs 72%), but previous self-harm was also better documented in 2015 (2.5% unknown) as compared with 2014 (9.7% unknown).

There were 105 episodes of repeat self-harm within 90 days of the index attendance in 2014 versus 97 episodes within the same time frame in 2015 (table 2). Including repeat episodes, the total number of ED attendances associated with index admissions in the first 3 months was 373 in 2014 and 381 in 2015. The average number of repeat episodes within 90 days relative to the index attendances decreased from 0.35 (105/298) in 2014 to 0.31 (97/318) in 2015.

**Figure 1:**
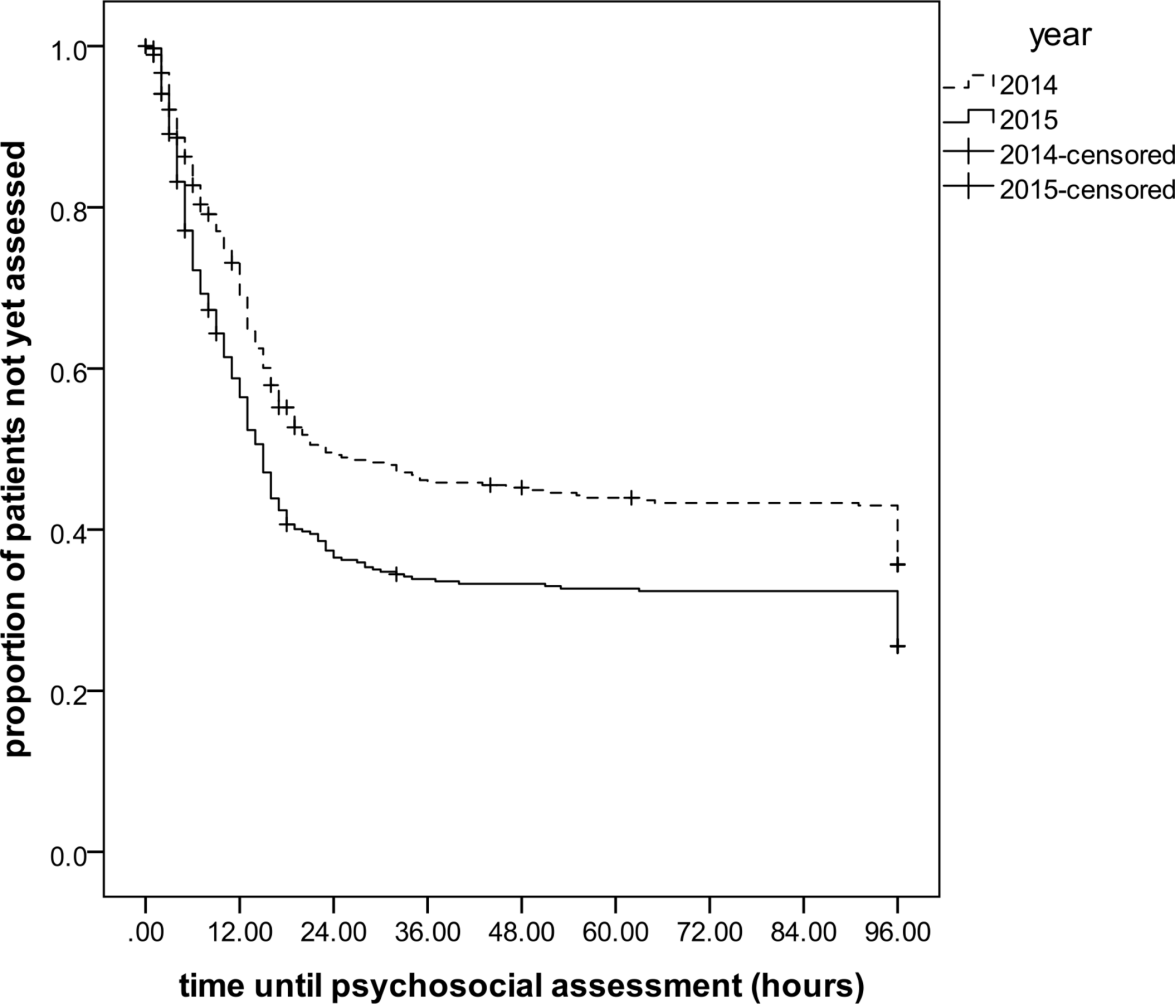
Time between arrival at emergency department and psychosocial assessment (log-rank test p value: 0.001).

With extended service hours in the LPS in 2015, the proportion of patients receiving a psychosocial assessment increased (from 57% to 68%; p=0.003; table 2). The proportion of patients receiving a psychosocial assessment outside 2014 LPS working hours increased from 29% in 2014 to 47% in 2015; p<0.001), and the median time between arrival at the ED and psychosocial assessment decreased by more than 3 hours (from 11 hours and 44 min to 8 hours and 30 min; p=0.017; figure 1). The median time between medical assessment and psychosocial assessment decreased by two and a half hours (from 9 hours and 30 min to 6 hours and 53 min), and this was also evident in the subgroup of patients attending during the original LPS office hours (from 10 hours and 20 min to 8 hours and 28 min; p=0.003). The proportion of episodes where patients were admitted to a ward slightly increased (from 68% to 69%); relatively more were admitted to an observation ward (from 58% to 63%) and less often to an ITU (from 2.5% to 0.5%). The median length of stay for patients admitted to a ward remained unchanged (1 days), but the average stay decreased somewhat from 1.7 (SD 4.1) days to 1.4 (SD 2.8) days, but statistical evidence for this difference was weak (p=0.26). The number of patients self-discharging before assessment and/or follow-up arrangements decreased (from 20% to 13%; p=0.022). In 2015, patients were more often referred to the crisis team and other community teams (increasing from 12.3% to 15.2% and from 7.2% to 15.2%, respectively), and less often to the self-harm clinic (decreasing from 15.3% to 4.7%).

**Table 2:** Differences in the process of care before and after the extended LPS

	January–March 2014 (n=373)	January–March 2015 (n=381)	Difference (abs)	p Value
Psychosocial assessment n (%)*	213 (57)	258 (68)	+45 (+11%)	0.003
Assessor for those who had a, n (%)*	(n=213)	(n=258)		
Psychiatrist	109 (51)	77 (30)	−32 (−21%)	<0.001
Liaison nurse	71 (33)	166 (64)	+95 (+31%)	
Other	24 (5.9)	13 (5.0)	−1 (−0.9%)	
Unknown	9 (5.9)	2 (0.8)	−10 (−5.1%)	
Psychosocial assessment by hour of day, n (%)*				
Monday to Friday 09:00–17:00 hours	133 (62)	117 (46)	−16 (−16%)	<0.001
All other times	61 (29)	121 (47)	+60 (+18%)	
Unknown	19 (8.9)	20 (7.8)	+1 (-1.1%)	
Median time from ED arrival to psychosocial assessment†	11 hours 44 min	8 hours 30 min	−3 hour 14 min	<0.017
Median time from medical assessment to psychological assessment†				
Overall	9 hours 30 min (n=185)	6 hours 53 min (n=230)	−2 hour 37 min	<0.001
Attendances between 09:00 and 17:00 hours	2 hours 51 min (n=44)	2 hours 59 min (n=60)	+8 min	0.078
Attendances during other hours	10 hours 20 min (n=141)	8 hours 28 min (n=170)	−1 hour 52 min	0.003
Admission to ward, n (%)*				
No	121 (32)	117 (31)	−4 (−1%)	0.110
Yes—observation ward	212 (58)	238 (63)	+26 (+5%)	
Yes—ITU	9 (2.5)	2 (0.5)	−7 (−2%)	
Yes—other	24 (6.6)	21 (5.6)	−3 (−1%)	
Median (p25–75) duration of hospital stay if not admitted (hours)	No data‡	12 (7–21)		
Mean duration of hospital stay if admitted (days)§	1.7 (4.1)	1.4 (2.8)	−0.37	0.26§
Median (p25–75) duration of hospital stay if admitted to ward/ITU (days)¶	1 (1–1)	1 (1–1)	=	0.004
Total admission days (including ITU)	480	393	−87	
Outcome of ED attendance, n (%)*				
Psychiatric inpatient admission	11 (2.9)	11 (2.9)	-/-	0.96
Crisis team	46 (12)	58 (15)	+12 (+3%)	0.25
Other community team	27 (7.2)	58 (15)	+31 (+8%)	0.001
Self-harm clinic	57 (15)	18 (4.7)	−39 (−10%)	<0.001
Alcohol nurse service	19 (5.1)	20 (5.2)	+1 (+0.1%)	0.92
Home/GP care only	94 (25)	89 (23)	−5 (−2%)	0.56
Social services	0 (0)	3 (0.8)	+3 (+0.8%)	0.086
Voluntary agency	0 (0)	13 (3.4)	+13 (+3.4%)	<0.001
Custody	25 (6.7)	14 (3.7)	−11 (−3%)	0.061
Died	0 (0)	1 (0.3)	+1 (+0.3%)	0.51
Patients self-discharging before a psychosocial assessment is carried out, n (%)*	73 (20)	51(13)	−22 (−7%)	0.022
Episodes with repeat self-harm<90 days, n (%)				
Total repeat episodes (min–max)**	105 (0–19)	97 (0–9)	−8 (−8%)	0.79
First repeat after index, n (%)	48 (18)	54 (17)	+6 (-1%)	
First episode of self-harm, n (%)	3 (5.6)	2 (4.3)	−1 (−1.3%)	
Previous self-harm, n (%)	45 (21)	52 (20)	+7 (−1%)	

*p Value based on Χ^2^ test.

†p Value based on log-rank test.

‡This element was not documented from the start of the self-harm register.

§p Value based on bootstrap corrected t-test statistic.

¶p Value based on Wilcoxon-Mann-Witney test.

**p Value based on Wilcoxon-Mann-Witney test for # attendances per patient.

††p Value based on Cox regression, adjusted for previous self-harm, sex and suicide risk.

ED, emergency department; ITU, intensive treatment unit; LPS, liaison psychiatric service.

**Figure 2:**
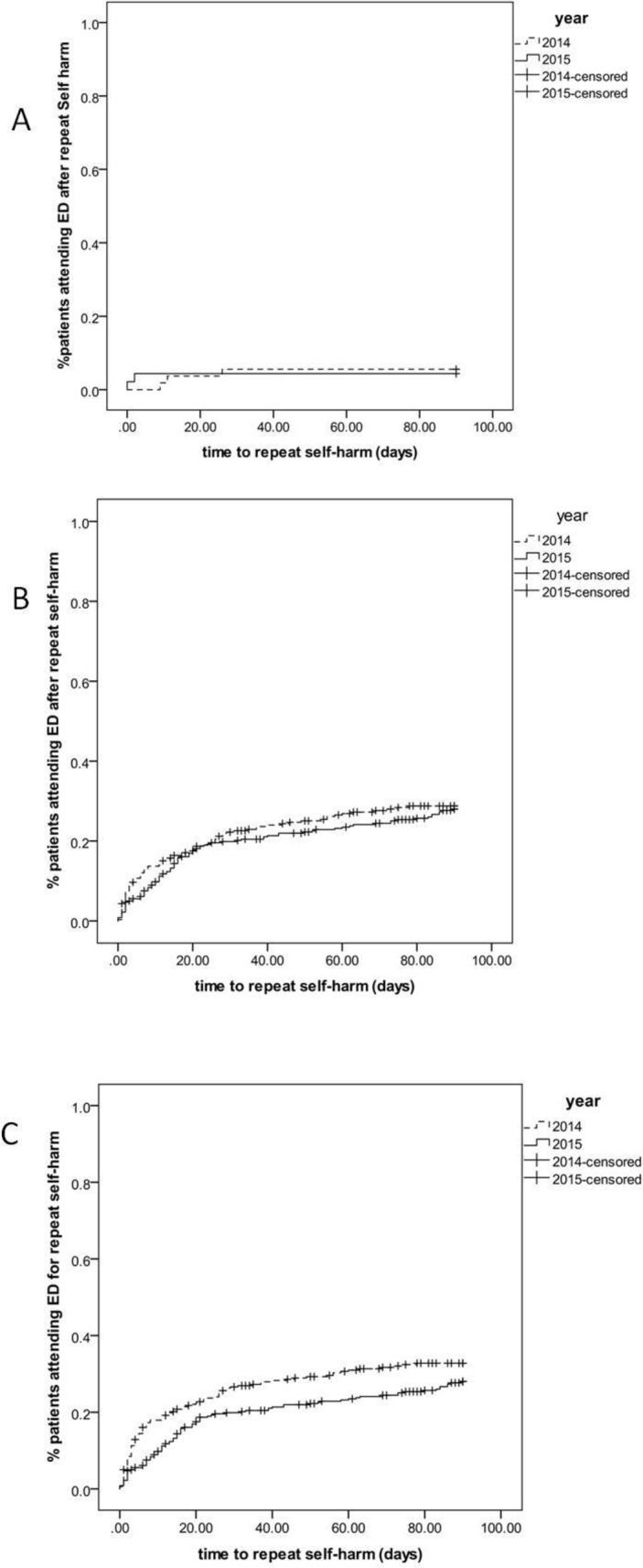
Cumulative incidence of repeat episodes of self-harm following the index attendance in patients with no previous self-harm (A); previous self-harm (B) and all episodes of repeat self-harm <90 days (C) for 2014 and 2015, based on Kaplan-Meier analyses. ED, emergency department.

There was no evidence of a reduction in repeat self-harm episodes following the introduction of extended LPS working hours either among patients with or without a previous self-harm episode (figure 2A and B). The Kaplan-Meier analysis indicated that there was some evidence of a reduction in the incidence of repeat self-harm within 90 days of the index episode in 2015 compared with 2014. Proportional hazards regression suggested that in 2015, patients were less likely to reattend the ED for self-harm within 90 days than those in the same period in 2014 (crude risk ratio 0.86; 95% CI 0.51 to 1.44; figure 2C) although statistical evidence for a difference was weak (p=0.56). In a model controlling for patient characteristics (previous self-harm, age, sex and matrix risk), this association appeared slightly stronger (adjusted risk ratio 0.79; 95% CI 0.47 to 1.33; p=0.37).

The average cost per attendance decreased from £784 in 2014 to £700 in 2015, a cost reduction of −11% per episode. However, the 95% CI around the mean difference was large (mean difference −£84; 95% Bootstrap CI −£254 to £77). The higher costs of more LPS assessments may be offset by reduced costs of ITU and ward bed days (table 3).

**Table 3:** Mean total costs per attendance for index patients in January–March 2014 and 2015 and repeated self-harm episodes within 90 days

Unit	Unit costs	2014		2015		Difference	
		(n=403 attendance*)		(n=415 attendance*)			
		Volume	Costs	Volume	Costs	Volume	Costs
ED attendance							
ED	£133.20	0.35	£46	0.32	£42	−0.03	−£4
ED+admission	£205.85	0.65	£134	0.68	£140	0.03	£6
LPS assessment	£187.45	0.55	£103	0.67	£126	0.12	£23
Subtotal ED attendances			£283		£309		£25 (£11 to £39)
Hospital admissions							
Observation ward (days)	£405.05	0.97	£391	0.83	£335	−0.1	−£56
ITU (days)	£1058.75	0.03	£29	0.01	£13	−0.02	−£16
Other ward (days)	£405.05	0.20	£80	0.11	£44	−0.09	−£36
Subtotal admissions			£500		£391		−£109 (−£276 to £50)
Mean total costs per attendance			£784		£700		−£84 (−£254 to £77)
Total costs			£315 843		£290 562		−£25 281

*Cost estimates are based on all attendances within 90 days after index attendance; figures may therefore differ slightly from those in table 2.

**95% bootstrap CI based on 1000 bias corrected accelerated bootstraps.

ED, emergency department; ITU, intensive treatment unit; LPS, liaison psychiatric service.

The average cost per patient (including repeat attendances within 90 days) decreased from £1060 in 2014 to £914 in 2015 (mean difference −£146; 95% BCI: −£433 to £138), a cost reduction of −14% per patient. The total costs for 298 patients attending the ED in the first 3 months of 2014 amounted to £315 843, whereas in the same period in 2015 the total costs for 318 patients attending the ED amounted to £290 562. Although more patients presented at the ED in 2015, the total costs associated with these self-harm episodes decreased by £25 281 (−8.0%). If extrapolated to a full year, this equates to savings of approximately £101 000 or £144 600 if the total is estimated excluding LPS assessment costs. This suggests that the annual investment (£250 000) in extending the LPS was associated with a cost reduction of £144 600 in the non-LPS hospital costs of care for patients presenting at the ED following self-harm despite the small increase in patient numbers.

Sensitivity analyses showed that the estimated cost per patient attending the ED was consistently lower in 2015 as compared with 2014, ranging from −£60 to −£107 (table 4). The impact on total costs amounted to savings between £68 300 and £133 900 per year.

**Table 4:** Sensitivity analyses: mean costs per attendance, difference between 2014 and 2015 and total cost impact for cohort for different assumptions and estimates

Analysis		Mean cost per attendance			Total cohort
ID	Description	2014	2015	Difference	Difference
0	Main analysis (base case)	£784	£700	−£84	−£25 281
1	Differentiate PS assessment by liaison nurse/psychiatrist	£783	£685	−£98	−£31 174
2	Assume observational unit costs for all bed days	£766	£692	−£74	−£21 328
3	Unit cost LPS assessment—low (−25%)	£758	£669	−£89	−£27 997
4	Unit cost LPS assessment—high (+25%)	£809	£732	−£78	−£22 565
5	Unit cost observational ward—low (−25%)	£666	£605	−£60	−£17 076
6	Unit cost observational ward—high (+25%)	£902	£795	−£107	−£33 486

ITU, intensive treatment unit; LPS, liaison psychiatric service; PS, psychosocial.

## Discussion

### Main findings

We compared two cohorts of patients attending the ED following self-harm during a 3-month period in 2014 and 2015, following a £250 000 investment to extend LPS operating hours. Clear improvements were found in the proportion of patients receiving a psychosocial assessment as well as the time between ED attendance and psychosocial assessment and reductions in self-discharge prior to assessment. There was a suggestion that the incidence of repeat self-harm declined, but we lacked statistical power to detect modest but clinically important effects. There was no evidence that the proportion of patients admitted to hospital decreased, however, the mean cost per patient (including repeat attendances) declined by approximately 14%. The findings from this analysis indicate that much of the additional £250 000 investment in liaison psychiatry services was offset by cost savings and improvements in management for self-harm patients. However, a larger study would be needed to confirm this.

The considerable decrease in referrals to the self-harm clinic and somewhat smaller increase in referrals to the crisis team probably reflects improved service delivery for self-harm patients, as people who were previously discharged without an assessment were offered follow-up at the self-harm clinic the next day or within a couple of days; whereas with higher levels of assessment, fewer people were referred to this clinic and more were referred to specialist mental health services.

### Strengths and limitations

Our study has several strengths. First, rather than simply estimating the cost of the care of people who have self-harmed,^16–18^ we have sought to estimate the impact of additional investment in liaison psychiatry services and whether investment in this area results in cost savings for example, arising from shorter periods of hospitalisation.

Second, our analysis was based on an unselected series of consecutive hospital presentations with self-harm and includes hospital admissions as well as ED attendances that did not lead to admission. Previous studies have been based on select patients groups, for example, those taking an overdose^8^ or on small numbers of patients.^19^

Lastly, we compared activity of the whole service rather than attempting to identify patients who would have received the service prior to its inception, the approach used in the analysis of the Rapid Assessment Interface and Discharge (RAID) liaison psychiatry service in Birmingham, UK.^11^

Nevertheless, there are a number of important limitations. Unlike the recent evaluation of the RAID service in Birmingham, UK, we did not measure the impact of other aspects of the psychiatric liaison team’s activity; assessment of people who self-harm comprises only 40% of LPS referrals in the hospital in our study and a smaller proportion in the RAID evaluation. It is likely that the increase in LPS operating hours will provide a better service for all hospitalised patients with psychiatric morbidity.

In addition, we did not assess the impact of extended LPS on the entire package of care following presentation. In contrast, Sinclair (2011) estimated costs based on longer term follow-up.^16^ In addition to service costs associated with psychiatric or community mental health services during follow-up, there may also be measurable patient benefits when using a longer time horizon, but possibly also increased costs resulting from increased identification of (and referral for) psychiatric/social problems.

Furthermore, though our analysis was based on over 300 presentations before and after the introduction of extended liaison services, we had insufficient statistical power to demonstrate clinically important differences in some outcomes. For instance, the high variance between patients in length of stay and costs limits our ability to reach definitive conclusions about these outcomes. Also the observed 20% reduction in repeat self-harm episodes would be clinically important, but lacked statistical robustness.

Lastly, this analysis does not include a comparison hospital to control for secular trends in mental health and service provision. Despite the study limitations the major step-change in care provided a good opportunity to evaluate the impact of a more accessible LPS service, while detailed and relatively complete individual level patient data from a local self-harm register facilitated accurate estimation of repeat self-harm and secondary care costs for self-harm patients.

### Findings in the context of the wider literature

Deficiencies and variations in the care of people presenting to hospital following self-harm have long been recognised.^7 8^ Almost 20 years ago Kapur et al. (1998) explored differences in service delivery between hospitals with and without self-harm teams.^7^ In general, the level of service provision was considerably lower than in our study; Kapur reported that in hospitals without a self-harm team 39% of patients received a psychosocial assessment whereas in hospitals with such a team the proportion receiving an assessment was 46%. In our study 57% of patients were assessed in 2014, rising to 67% in 2015.

Due to different costing methods, cost implications are more difficult to compare across studies. Cost savings reported by Tadros *et al* evaluating the impact of the Birmingham RAID service were in the range of £3.4 to £9.5 million a year.^20^ These estimates are based on a comparison of lengths of stays and rates of readmission only, and most of these savings come from reduced bed use among elderly patients. Our study only focused on LPS activities for self-harm patients, and potential benefits to and savings from care to elderly patients thus have not been included.

Sinclair *et al* examined economic findings based on a different approach, examining patterns of resource use and costs over a 7-year follow-up period and estimated how different factors contribute to total costs using regression analyses.^16^ Average costs were £2944 (SD £8438) per patient. Their study clearly illustrated the long-term costs associated with the provision of health and social care to this patient group as social and mental health problems generally persist after episodes of crisis and are largely managed by community health services. Appropriate and effective pathways of care thus generate additional costs for self-harm patients but also provide opportunity for more efficient, that is, cost-saving solutions.

The only study that seems to allow direct comparison of cost figures is by Kapur *et al*, reporting costs associated with hospital admission following deliberate self-poisoning, comparing hospitals with and without self-harm teams.^18^ Although they evaluated different service models, their estimates for hospital-related costs were largely comparable (£510 vs £390 for hospitals with and without self-harm teams and £500 vs £391 for self-harm attendances before and after extension of service hours, respectively). They also demonstrated potential costs savings to be achieved by investing in more appropriate services for self-harm patients, although the particular interventions being compared were different.

Opportunities to improve healthcare and outcomes for self-harm patients also lie in identifying/developing effective psychiatric interventions for individual patients. For example, there is good evidence from systematic reviews that cognitive behavioural therapy-based interventions reduce the incidence of repeat self-harm by almost one third.^21^ Patients benefiting from such interventions are likely to use less medical and community health services, and effective interventions thus have a high likelihood to be also cost-effective.^22^ As patients who frequently repeat self-harm generate the highest costs,^16^ psychiatric interventions reducing the risk of repeat self-harm may be cost saving on the longer term, as demonstrated for cognitive behavioural therapy.^22^

### Implications

Improving the quality of care in health services and investing in preventative services such as LPS is a difficult and continuous challenge, especially at a time of economic austerity. However, the government’s mental health strategy recognises the need for improved services at the interface between mental and physical health.^23^ Our evaluation emphasises the importance of protecting and even expanding services where there is good evidence of improved clinical outcomes. It also highlights the need to adequately evaluate changes in healthcare models and associated costs before they are widely implemented.^24^ Increasing pressures on NHS budgets mean there is a danger of cutting funding for services that can potentially save very significant amounts of money for the local health economy in the long term. Apart from such financial constraints, the next question would be whether there are sufficient staff numbers available with the relevant skills and expertise to fill these roles.

There is limited evidence of the cost-effectiveness of liaison psychiatry services; further work is needed in this area. In addition to health technology assessments evaluating (cost-)effectiveness of interventions for individual self-harm patients, high-quality cluster randomised trials are needed to reduce uncertainty regarding what are the most effective models of care for people experiencing mental health crisis.

## Supplementary Material

Reviewer comments

Author's manuscript

## References

[1] Hawton K, Bergen H, Casey D, et al Self-harm in England: a tale of three cities. Multicentre study of self-harm. Soc Psychiatry Psychiatr Epidemiol 2007;42:513–21. 10.1007/s00127-007-0199-7 17516016

[2] Cooper J, Steeg S, Bennewith O, et al Are hospital services for self-harm getting better? An observational study examining management, service provision and temporal trends in England. BMJ Open 2013;3:e003444 10.1136/bmjopen-2013-003444 PMC3840333 24253029

[3] Carroll R, Metcalfe C, Gunnell D Hospital presenting self-harm and risk of fatal and non-fatal repetition: systematic review and meta-analysis. PLoS One 2014;9:e89944 10.1371/journal.pone.0089944 24587141 PMC3938547

[4] Hawton K, Zahl D, Weatherall R Suicide following deliberate self-harm: long-term follow-up of patients who presented to a general hospital. Br J Psychiatry 2003;182:537–42. 10.1192/bjp.182.6.537 12777346

[5] Zahl DL, Hawton K Repetition of deliberate self-harm and subsequent suicide risk: long-term follow-up study of 11,583 patients. Br J Psychiatry 2004;185:70–5. 10.1192/bjp.185.1.70 15231558

[6] CG16 Self-harm: the short-term physical and psychological management and secondary prevention of self-harm in primary and secondary care: National Institute for Clinical Excellence (NICE), 2004 https://www.nice.org.uk/guidance/cg16/evidence/full-guideline-189936541 21834185

[7] Kapur N, House A, Creed F, et al Management of deliberate self poisoning in adults in four teaching hospitals: descriptive study. BMJ 1998;316:831–2. 10.1136/bmj.316.7134.831 9549454 PMC28487

[8] Kapur N, House A, Dodgson K, et al Effect of general hospital management on repeat episodes of deliberate self poisoning: cohort study. BMJ 2002;325:866–7. 10.1136/bmj.325.7369.866 12386037 PMC129633

[9] Carroll R, Metcalfe C, Steeg S, et al Psychosocial assessment of self-harm patients and risk of repeat presentation: an instrumental variable analysis using time of hospital presentation. PLoS One 2016;11:e0149713 10.1371/journal.pone.0149713 26918579 PMC4769277

[10] Bennewith O, Gunnell D, Peters T, et al Variations in the hospital management of self harm in adults in England: observational study. BMJ 2004;328:1108–9. 10.1136/bmj.328.7448.1108 15130979 PMC406323

[11] Parsonage M, Fossey M Economic evaluation of a liaison psychiatry service: Centre of Mental Health, 2011 https://www.centreformentalhealth.org.uk/evaluation-liaison-psychiatry

[12] Craig P, Cooper C, Gunnell D, et al Using natural experiments to evaluate population health interventions: guidance for producers and users of evidence: Medical Research Council (MRC), 2010 https://www.mrc.ac.uk/documents/pdf/natural-experiments-guidance/ 10.1136/jech-2011-200375 PMC3796763 22577181

[13] Gilbody S, House A, Owens D The early repetition of deliberate self harm. J R Coll Physicians Lond 1997;31:171–2. 9131517 PMC5420906

[14] Fitzmaurice GM, Laird NM, Ware JH Applied longitudinal analysis. Second Edition Hoboken, New Jersey: John Wiley & Sons, 2011.

[15] NHS reference costs 2014 to 2015: Department of Health (DH), 2015 https://www.gov.uk/government/publications/nhs-reference-costs-2014-to-2015

[16] Sinclair JM, Gray A, Rivero-Arias O, et al Healthcare and social services resource use and costs of self-harm patients. Soc Psychiatry Psychiatr Epidemiol 2011;46:263–71. 10.1007/s00127-010-0183-5 20174782

[17] Shepard DS, Gurewich D, Lwin AK, et al Suicide and suicidal attempts in the United States: costs and policy implications. Suicide Life Threat Behav 2016;46:352–62. 10.1111/sltb.12225 26511788 PMC5061092

[18] Kapur N, House A, Dodgson K, et al Management and costs of deliberate self-poisoning in the general hospital: a multi-centre study. J Ment Health 2002;11:223–30. 10.1080/1-09638230020023606

[19] Sgobin SM, Traballi AL, Botega NJ, et al Direct and indirect cost of attempted suicide in a general hospital: cost-of-illness study. Sao Paulo Med J 2015;133:218–26. 10.1590/1516-3180.2014.8491808 26176926 PMC10876379

[20] Tadros G, Salama RA, Kingston P, et al Impact of an integrated rapid response psychiatric liaison team on quality improvement and cost savings: the Birmingham RAID model. Psychiatrist 2013;37:4–10. 10.1192/pb.bp.111.037366

[21] Hawton K, Witt KG, Salisbury TL, et al Psychosocial interventions following self-harm in adults: a systematic review and meta-analysis. Lancet Psychiatry 2016;3:740–50. 10.1016/S2215-0366(16)30070-0 27422028

[22] Byford S, Knapp M, Greenshields J, et al Cost-effectiveness of brief cognitive behaviour therapy versus treatment as usual in recurrent deliberate self-harm: a decision-making approach. Psychol Med 2003;33:977–86. 10.1017/S0033291703008183 12946082

[23] The mental health strategy for England - No Health without Mental Health: a cross-government mental health outcomes strategy for people of all ages: Department of Health (DH), 2011 https://www.gov.uk/government/uploads/system/uploads/attachment_data/file/213761/dh_124058.pdf

[24] Sinclair JM, Gray A, Hawton K Systematic review of resource utilization in the hospital management of deliberate self-harm. Psychol Med 2006;36:1681–93. 10.1017/S0033291706008683 16938143

