# Supplementary material for: Extending the liaison psychiatry service in a large hospital in the UK: a before and after evaluation of the economic impact and patient care following ED attendances for self-harm
Source: BMJ Open. 2017 Aug 21;7(8):e016906. doi: 10.1136/bmjopen-2017-016906 (PMC5724115; doi:10.1136/bmjopen-2017-016906)
Supplement: Author's manuscript [file bmjopen-2017-016906.draft_revisions.pdf]

BMJ Open is committed to open peer review. As part of this commitment we make the peer review history of every article we publish publicly available.

When an article is published we post the peer reviewers' comments and the authors' responses online. We also post the versions of the paper that were used during peer review. These are the versions that the peer review comments apply to.

The versions of the paper that follow are the versions that were submitted during the peer review process. They are not the versions of record or the final published versions. They should not be cited or distributed as the published version of this manuscript.

BMJ Open is an open access journal and the full, final, typeset and author-corrected version of record of the manuscript is available on our site with no access controls, subscription charges or pay-per-view fees (http://bmjopen.bmj.com).

If you have any questions on BMJ Open's open peer review process please email info.bmjopen@bmj.com

BMJ Open

Extending the liaison psychiatry service: a before and after evaluation of the economic impact in Emergency Department attendances for self-harm

Journal:	*BMJ Open*
Manuscript ID	bmjopen-2017-016906
Article Type:	Research
Date Submitted by the Author:	21-Mar-2017
Complete List of Authors:	Opmeer, Brent; University of Bristol, School of Social and Community Medicine; NIHR Collaboration for Leadership in Applied Health Research and Care West<br>Hollingworth, William; University of Bristol, School of Social and Community Medicine<br>Marques, Elsa; University of Bristol, School of Clinical Sciences<br>Margelyte, Ruta; University of Bristol, School of Social and Community Medicine; NIHR Collaboration for Leadership in Applied Health Research and Care West,<br>Gunnell, David; Bristol University, School of Social and Community Medicine
**Primary Subject Heading**:	Health services research
Secondary Subject Heading:	Health economics, Mental health
Keywords:	Suicide & self-harm < PSYCHIATRY, psychosocial assesement, service evaluation, ACCIDENT & EMERGENCY MEDICINE, liaison psychiatry

SCHOLARONE™ Manuscripts

For peer review only

Title

Extending the liaison psychiatry service: a before and after evaluation of the economic impact in Emergency Department attendances for self-harm

Authors

BC Opmeer[1,2,3], W. Hollingworth[1,2], E. Marques[4] , R. Margelyte[1,2], D. Gunnell[1]

Author affiliations:

[1] School of Social and Community Medicine, University of Bristol, UK
[2] The National Institute for Health Research Collaboration for Leadership in Applied Health Research and Care (CLAHRC) West at University Hospitals Bristol NHS Foundation Trust, Bristol, UK
[3] Clinical Research Unit, Academic Medical Centre, Amsterdam, the Netherlands
4 School of Clinical Sciences, University of Bristol, UK

Corresponding author

Dr Brent Opmeer, NIHR CLAHRC West at University Hospitals Bristol NHS Foundation Trust, 9th Floor, Whitefriars, Lewins Mead, Bristol BS1 2NT, UK. bo15082@bristol.ac.uk, phone: +31-624 548 900

For peer review only

For peer review only - http://bmjopen.bmj.com/site/about/guidelines.xhtml

ABSTRACT

Objectives: To evaluate the impact of an expansion of Liaison Psychiatry Services (LPS) on patient management, outcomes and treatment costs for Emergency Department (ED) attendances for self-harm.

Design: Retrospective before and after cohort study using routinely collected Self-Harm Surveillance Registry data.

Setting: A large hospital in South West England.

Subjects: Patients attending the ED for self-harm.

Interventions: Extension of the Liaison Psychiatry Service's operating hours from 9am to 5pm Monday to Friday to 8am to 10pm 7 days a week, following a £250,000 annual investment

Main outcome measures: Number and characteristics of ED attendances for self-harm. The before and after cohorts were compared in terms of key process measures, including proportion of patients receiving a psychosocial assessment, average length of hospital stay, waiting times for assessment, proportion of patients who self-discharged without an assessment, levels of repeat self-harm attendances and mean cost per patient attendance.

Results: 298 patients attended ED for self-harm on 373 occasions between January and March 2014, and 318 patients attended on 381 occasions between January and March 2015. The proportion of ED attendances where patients received a psychosocial assessment increased from 57% to 68% ($p<0.005$), median waiting time decreased by 3h14min ($p<0.05$), and the proportion of episodes where patients self-discharged without a psychosocial assessment decreased from 20% to 13% ($p < 0.05$). The mean cost per patient attendance was marginally lower after the intervention (-£84; 95%CI: -£254 to £77).

Conclusions: The extended liaison psychiatry services seems to have a favourable effect on the management and service outcomes of slef-harm patients. The additional cost might be partially offset by more efficient assessment and discharge. The wider impact of extended LPS on other hospitalised patients would require further evaluation.

For peer review only - http://bmjopen.bmj.com/site/about/guidelines.xhtml

SUMMARY

Strengths:

- There was a major step-change in care provision (increase in service availability from 40 to 98 hours per week), providing a good opportunity to evaluate the impact of changed service provision

- Detailed and relatively complete individual level patient data were available from a bespoke self-harm register, facilitating estimation of resource costs for self-harm patients.

Limitations:

- Analysis does not assess the wider impact of extended LPS on post-discharge service provision, and patients with other mental health conditions seen by the LPS.

- Analysis does not include a control site for wider control of economic, social and political trends in mental health and service provision.

- Analysis of two relatively short evaluation periods has implications for sample size and associated power, and does not enforce even distribution in first and repeat self-harm episodes.

For peer review only

For peer review only - http://bmjopen.bmj.com/site/about/guidelines.xhtml

INTRODUCTION

There are an estimated 200,000 Emergency Department (ED) attendances for self-harm in England & Wales every year(1) and approximately half of these result in admission to a hospital ward(2). Self-harm is often repeated, with more than 15% of individuals who attend a hospital with self-harm re-attending within a year(3). A history of self-harm is the main risk factor for suicide across a range of psychiatric disorders(4). Repeated self-harm further increases suicide risk(5) Providing effective, evidence based clinical care for this high-risk patient population is a key of reducing their risk of subsequent self-harm and suicide.

UK clinical guidelines suggest that all patients should be offered a preliminary psychosocial assessment after an act of self-harm to determine mental capacity, willingness to remain for further assessment, level of distress and the possible presence of mental illness(6). However in many UK hospitals, more than half of patients are discharged from the emergency department without the assessment(2). Patients who leave the ED without a psychosocial assessment are less likely to be offered a follow-up(7). There is also evidence that psychosocial assessments reduce risk of repeat self-harm(8, 9).

Liaison Psychiatry Services (LPS) have been introduced to hospitals to provide assessment and care for patients presenting to the ED with mental health problems and to support people with physical health problems who also have or develop mental health problems such as delirium. Despite the existing guidelines on the management of self-harm(6), there are significant variations in service models in terms of staffing, coverage or management(2, 10). Most people who have self-harmed seek help at times when only an emergency mental health service is available. One response is to invest in extended LPS operating hours, but the benefits of such investment have not been well established. An exception is the economic evaluation of expanded LPS service at a large acute hospital in Birmingham which found that additional investment in the services generated incremental benefits in terms of reduced bed use with overall benefit to cost ratio of more than 4:1 (11).

In 2014, a local Clinical Commissioning Group (CCG) invested approximately £250,000 per annum in an extended LPS at a large teaching hospital with a consultant-led 24hour ED. This was used to increase the working hours of the LPS from Monday to Friday 0900-1700 (i.e. 40 hours per week) to seven days a week 0800-2200 (98 hours per week). To achieve this increase in service provision, four additional full time liaison nurses were employed. The aim was to increase the proportion of patients attending the emergency department after self-harm who receive a psychosocial assessment and reduce admissions to acute hospital beds to await LPS assessment. It was anticipated that these changes might also lead to better patient outcomes such as reduced repeat self-harm and suicide as a result of increases in the proportion of patients receiving appropriate follow-up care.

For peer review only - http://bmjopen.bmj.com/site/about/guidelines.xhtml

Our primary objective is to assess the impact of the extended LPS on process measures and indicators of patient outcomes and costs following self-harm. We assess resource use and costs associated with management of patients attending the emergency department following self-harm. Our findings will enable commissioners to explore whether extra investment in LPS can improve patient management and outcomes of self-harm and result in cost savings.

For peer review only

For peer review only - http://bmjopen.bmj.com/site/about/guidelines.xhtml

METHODS

Study design

The change in LPS provision is a natural experiment(12) whereby there has been a step-change in the availability of a service from a defined point in time (1st of September 2014). We compared the process, cost and outcomes of care for patients attending the ED following self-harm in a before and after study. We estimated NHS secondary care costs, although we recognise that care provide by the LPS will have spill-over effects on primary and community care services.

Participants and data

We compared two patient cohorts presenting following self-harm at the ED in January 1st – March 31st 2014 (Before) and Jan 1st – March 31st 2015 (After). Our focus is on self-harm patients because these are the group of psychiatric patients most likely to be admitted to a hospital bed, and was the focus of the commissioner's new funding. However, it is important to note that self-harm patients comprise only 40% of the LPS workload, therefore the extended service will have an impact on a wider group of patients.

Patients were identified from the local Self-Harm Surveillance Register (SHSR). The SHSR was established in 2010 and records clinical and sociodemographic details of all hospital-presentations for self-harm at the hospital. Using the SHSR we identified the index self-harm ED attendance in each period (i.e. the first time a patient attended between Jan 1st and March 31st) and any repeat self-harm ED attendance within a 90 day period of the index episode. Data collection is approved by the Central Bristol Research Ethics Committee. We selected time periods which are not adjacent to the service change date (September 2014) to avoid the period when the service might have been 'ramping up' or 'bedding in'. We selected the same 3 calendar months for the before and after periods to avoid bias due to seasonal trends in mental health and self-harm. The follow-up period was defined on the basis that a high proportion of all patients who repeat self-harm within 12 months do so within 90 days of the index event(13).

Data quality/missing data

Data extracted from the SHSR were checked for inconsistencies, and where evident resolved. Audits reveal SHSR case ascertainment is >95%.

Outcomes

Key characteristics to describe the ED attendance for self-harm include type of self-harm, whether patient has previously self-harmed, and matrix risk assessment. The matrix is a locally developed tool for use by ED to determine the urgency with which a patient should be referred for psychosocial assessment. Patients are categorised into three groups (red, amber or green) depending on the degree of urgency.

Process measures of interest are the proportion of patients receiving a psychosocial assessment; waiting times from attendance to assessment; proportion of patients self-discharging from the ED without assessment; proportion of episodes admitted to a ward; and average length of hospital stay. Patient outcome measures include the proportion of patients with repeat self-

For peer review only - http://bmjopen.bmj.com/site/about/guidelines.xhtml

harm ED attendances; number of repeat self-harm ED attendances; and time to repeated self-harm attendance. We also evaluated change in mean cost per self-harm ED attendance as well as mean cost per patient associated with the index self-harm episode (including repeat self-harm episodes within 90 days).

Statistical analyses

Analyses describing characteristics of patients attending the ED for self-harm are based on index attendances during the three-month periods (January-March) in 2014 and 2015. Analyses describing the evaluation of impact on service delivery are based on total number of attendances in the three months (including repeat attenders) in 2014 and 2015. Analyses describing the impact on risk of repeat self-harm are based on index attendances and all subsequent attendances for repeat self-harm within 90 days associated with these index attendances (i.e. including attendances up to June).

Descriptive analyses

Patient characteristics of the study population by year are reported descriptively. Continuous variables are summarized as means and standard deviations, for categorical variables, the number and percentage of participants/attendances within each category are presented.

Evaluation of impact

Differences in the process of care are examined for attendances before and after the extended LPS became operational. Proportions and medians for service outcome measures by year are reported with the absolute difference between the years and associated p-values (two tailed chi-square or t-test). This includes the proportion of attendances that received psychosocial assessment within or outside LPS service hours and by assessor type, the median times from ED arrival or medical assessment to psychosocial assessment, the proportion of attendances admitted to general ward or intensive therapy unit (ITU) and the median duration of hospital stay after admission. As some patients were known to be (also) admitted to an ITU, but of unknown duration, we assumed this duration to be 30% of the total stay, with a minimum of 1 day, for these patients. The proportion of self-discharged cases and number of episodes of repeated self-harm within 90 days are also considered as outcomes of the LPS activities.

Kaplan Meier analyses were used to compare differences in time between ED arrival and psychosocial assessment before and after extended LPS.

Differences in time until repeat self-harm attendances within 90 days were also compared for 2014 and 2015 using Kaplan-Meier analyses and proportional hazards regression to adjust for relevant factors (previous self-harm, sex, and Matrix risk) associated with re-attendance rates. Time to repeat self-harm was compared before and after the extended LPS for index episodes of patients with and without previous self-harm, and overall for consecutive repeat self-harm episodes. We used an analytic approach that allowed for multiple repeat self-harm episodes during the 90 days follow-up, and used the robust "sandwich"

For peer review only - http://bmjopen.bmj.com/site/about/guidelines.xhtml

estimator to account for correlated observations within the same patient (i.e. that some patients are more likely to repeat self-harm than others)(14).

Mean cost per attendance was presented for the before and after period including index presentation and 90 days follow-up. All unit costs were estimated from the 2014/15 NHS reference costs(15). NHS reference costs for ED care are higher for patients subsequently admitted to a hospital bed (mean = £205.85) than for patients who are discharged from the ED (mean = £133.20). The NHS reference cost for an emergency department mental health liaison contact (£187) does not distinguish between those conducted by psychiatrists or nursing staff. We used this figure in our analysis, but the actual cost may be lower for assessments conducted by nurses. The cost of inpatient care will depend on the ward type, the type of treatment required and the length of stay. Any reductions in hospital admissions due to the extended LPS may be due to fewer short stay admissions to observation units or other wards while waiting for psychosocial assessment. Therefore, we used the average unit cost (£405.50) for a non-elective short stay admission for 'observation or counselling' as a proxy for the per diem cost of observational unit or other ward care. We used the average daily cost (£1,058.75) of adult medical critical care patients to estimate costs for ITU days. Bootstrapping was used to estimate 95% bootstrap confidence intervals for cost estimates. Robustness of the findings is assessed in several univariate sensitivity analyses. We varied unit cost estimates for ward admissions and LPS assessments. In addition, impact on the results of differentiating between LPS referrals where psychosocial assessment was carried out by a liaison nurse or by a psychiatrist. and of ignoring that some of the patients admitted to a ward were (also) admitted to an Intensive Treatment Unit.

Data preparation, tables, figures and analyses are documented and performed using statistical software SPSS version 23, and SAS version 9.4.

For peer review only - http://bmjopen.bmj.com/site/about/guidelines.xhtml

RESULTS

Between January and March 2014, 298 patients attended the ED following self-harm, whereas during the same months in 2015, 318 patients attended the ED following self-harm, an increase of 7%. (Table 1).
Only around 20% of ED attendances in 2015 occurred during the original LPS working hours (Mon-Fri 0900-1700).

Although there are some differences in the distribution of these patient characteristics, the two cohorts seem to be relatively similar. In 2015 slightly more women attended following self-harm (63% vs 57%), and fewer patients were unemployed (46% vs 57%). Slightly more patients were known to have a history of self-harm (83% vs 72%), but previous self-harm was also better documented in 2015 2.5% unknown) as compared to 2014 (9.7% unknown). In 2014, relatively more attendances were after self-poisoning (66%) and fewer self-injury (20%) as compared to 2015 (59% poisoning and 25% self-injury).

There were 105 episodes of repeat self-harm within 90 days of the index attendance in 2014, versus 97 episodes within the same timeframe in 2015. Including repeat episodes, the total number of ED attendances associated with index admissions in the first three months increased by 2.1% from 373 in 2014 to 381 in 2015. Thereby the average number of repeat episodes within 90 days relative to the index attendances decreased from 0.35 (105/298) to 0.31 (97/318).

<<INSERT TABLE 1 APPROX HERE>>

With extended service hours in the LPS in 2015, the proportion of patients receiving a psychosocial assessment increased (from 57% to 68; Table 2). The proportion of patients receiving psychosocial assessment outside 2014 LPS working hours increased from 28% in 2014 to 47% in 2015), and the median time between arrival at the ED and psychosocial assessment decreased by more than 3 hours (from 11h44m to 8h30m; Figure 1). The median time between medical assessment and psychosocial assessment decreased by 2 and a half hours (from 9h30m to 6h53m), and this was also evident in the subgroup of patients attending the original LPS office hours (from 10h20m to 8h28min). The proportion of episodes where patients were admitted to a ward slightly increased (from 68% to 69%), but relatively more to an observation unit (from 58% to 63%), and less often to an ITU (from 2.5% to 0.5%). The median length of stay of patients admitted to a ward remained unchanged (1 days), but the average stay slightly decreased from 1.7 (SD 4.1) days to 1.4 (SD 2.8) days. Also the number of patients self-discharging before assessment and/or follow-up arrangements decreased (from 20% to 13%). In 2015 patients were more often referred to the intensive mental health team and especially other community teams (from 0% to 2.1% and from 7.2% to 15% respectively), and less often to the self-harm clinic (from 15% to 4.7%).

For peer review only - http://bmjopen.bmj.com/site/about/guidelines.xhtml

<<INSERT TABLE 2 APPROX HERE>>

There is no apparent reduction observed in repeat self-harm episodes after the index ED attendance for patients without or with a previous self-harm episode (Figures 2A and 2B). Comparing all episodes between ED attendances within 90 days, the Kaplan-Meier analysis reflects a decreased repeat self-harm rate in 2015 as compared to 2014. Proportional hazards regression, taking into account that some patients are more likely to repeat self-harm than others, suggested that in 2015, patients were 20% less likely to re-attend the ED for self-harm within 90 days than those in the same period in 2014 (crude risk ratio 0.79; 95%CI: 0.47 to 1.32; Figure 2C) although evidence for a difference was weak (p = 0.37). The impact on repeat self-harm rate seemed to be even more pronounced when factors known to be predictive for repeat self-harm were taken into account (adjusted risk ratio 0.72; 95%CI: 0.43 to 1.23; p = 0.23).

The average cost per attendance decreased from £784 in 2014 to £700 in 2015. However, the 95% CI around the mean difference was large (mean difference-£84; 95% BCI: -£254 to £77). The higher costs of more LPS assessments may be offset by reduced costs of ITU and ward bed days (Table 3).

The average cost per patient with an index attendance during Jan-Mar (and including repeat attendances within 90 days) decreased from £1060 in 2014 to £914 in 2015 (mean difference -£146; 95% BCI: -£433 to £138), a cost reduction of -14% per patient). The total costs for 298 patients attending the ED in the first three months of 2014 amounted to £315,843, whereas in the same period in 2015 the total costs for 318 patients attending the ED amounted to £290,562. Although more patients presented at the ED in 2015, the total costs associated with these self-harm episodes decreased by £25,281 (-8.0%). If extrapolated to a full year, this equates to savings of approximately £101,000 or £144,600 if the total is estimated excluding LPS assessment costs. This suggests that the annual investment (£250,000) in extending the LPS was associated with a cost reduction of £144,600 in the non-LPS hospital costs of care for patients presenting at the ED following self-harm despite the small increase in patient numbers.

<<INSERT TABLE 3 APPROX HERE>>

Sensitivity analyses showed that the estimated cost per patient attending the ED was consistent lower in 2015 as compared to 2014, ranging from -£60 to -£107 (table 4). The impact on total costs amounted to savings between £17,076 and £33,486 for these cohorts, or £68,300 and £133,900 per year .

<<INSERT TABLE 4 APPROX HERE>>

For peer review only

For peer review only - http://bmjopen.bmj.com/site/about/guidelines.xhtml

DISCUSSION

We compared two cohorts of patients attending the ED following self-harm during a 3-month period in 2014 and 2015, following a £250,000 investment to extend LPS operating hours. Clear improvements were found for the proportion of patients receiving a psychosocial assessment as well as the time between ED attendance and psychosocial assessment, and likely a decrease in the re-attendance rate within 90 days following the first attendance in these periods. There was also a decrease in the proportion of patients self-discharging. After adjusting for previous self-harm and other prognostic variables; patients attending the ED after self-harm in 2015 were 13% less likely to repeat self-harm as compared to those in 2014, although the findings are disproportionally driven by the few frequent attenders, and larger samples are required for more robust statistical evidence. Mean cost per patient (including repeat attendances) declined by approximately 14%. The findings from this analysis indicate that much of the additional £250,000 investment in liaison psychiatry services was offset by cost savings and improvements in management for self-harm patients. However, a larger study with higher statistical precision would be needed to confirm this.

Our analysis does not assess the wider impact of extended LPS on other patients. The LPS receives referrals from the ED and inpatient wards for a range of mental health conditions. Self-harm only comprises around 40% of referrals at this hospital. It is likely that the increase in LPS operating hours will provide a better service for all patients with psychiatric morbidity at the hospital. Second we did not assess the impact of extended LPS on post-discharge service provision (e.g. community mental health services). The increase in referrals to the intensive mental health and other community teams would result in additional service costs and benefits for patients. Third, the number of patients/attendances included in the analyses is limited, therefore we have insufficient statistical power to demonstrate relevant differences in some outcomes. For instance, the high variance between patients in length of stay and costs limits our ability to reach definitive conclusions about these outcomes. Lastly, this analysis does not include a comparison hospital to control for temporal trends in mental health and service provision. Despite the study limitations the major step-change in care provided a good opportunity to evaluate the impact of a more accessible LPS service, while detailed and relatively complete individual level patient data from a local self-harm register facilitated accurate estimation of repeat self-harm and secondary care costs for self-harm patients.

Improving the quality of care in health services and investing in preventative services such as LPS is a difficult and continuous challenge especially at a time of economic austerity. However, the Government's mental health strategy recognises the need for improved services at the interface between mental and physical health(16). Our evaluation emphasizes the importance of protecting and even expanding services where there is good evidence of improved clinical outcomes. It also highlights the danger of cutting funding for services that can

For peer review only - http://bmjopen.bmj.com/site/about/guidelines.xhtml

potentially save very significant amounts of money for the local health economy in the long term.

Potential benefits of liaison psychiatry services are based on limited evidence. Further work is needed to examine the clinical and cost effectiveness of various aspects of emergency and inpatient care on service user outcomes. High-quality cluster randomized trials are needed to reduce uncertainty regarding what are the most effective models of care for people experiencing mental health crisis.

For peer review only

For peer review only - http://bmjopen.bmj.com/site/about/guidelines.xhtml

ACKNOWLEDGEMENTS/FUNDING

The Bristol Self-harm Surveillance Register is funded by AWP NHS Trust and Bristol City Council. We thank all members of University Hospitals Bristol NHS Foundation Trust's Liaison Psychiatry team, in particular Salena Williams (team leader) and Kat Bramley and Peter Kennedy-Chapman (data collection) for their role in data collection.

The research was supported by the National Institute for Health Research (NIHR) Collaboration for Leadership in Applied Health Research and Care West (CLAHRC West) at University Hospitals Bristol NHS Foundation Trust. The views expressed are those of the author(s) and not necessarily those of the NHS, the NIHR or the Department of Health.

CONTRIBUTORSHIP

Dr Brent Opmeer contributed to the writing and critical reviewing of the manuscript, development of methods, analysis of data, interpretation of results, consensus meetings;

Dr Elsa Marques contributed to the design of the evaluation, acquisition and analysis of data, the writing and critical reviewing of the manuscript;

Ruta Margelyte contributed to the drafting, writing and critical reviewing of the manuscript;

Prof William Hollingworth and Prof David Gunnell conceived the project and contributed to the design of the evaluation, development of methods, analysis of data, interpretation of results, and writing and critical reviewing of the manuscript.

Each author listed on the manuscript has seen and approved the submission of this version of the manuscript and takes full responsibility for the manuscript.

COMPETING INTERESTS

We have read and understood BMJ Open policy on declaration of interests and declare that we have no competing interests.

DATA SHARING

Research data set is available on request for verification of the data analyses.

For peer review only

TABLES

Table 1 Number and characteristics of patients index episodes of ED SH attendances in 2014 and 2015 periods*

	Jan - Mar 2014 (n=298)	Jan - Mar 2015 (n = 318)
Attendances by hour of day n (%)		
Monday to Friday 0900-1700	70 (21)	75 (24)
Other	228 (79)	242 (76)
Female n (%)	166 (57)	201 (63)
Age on years mean (SD)	34 (14)	35 (15)
Marital status n (%)		
Single	220 (74)	253 (80)
Married	30 (10)	32 (10)
Other	38 (13)	26 (8)
NK	10 (3)	7 (2)
Occupational status n (%)		
Employed	56 (19)	56 (18)
Unemployed	171 (57)	147 (46)
Other	55 (17)	80(25)
NK	14 (5)	35 (11)
Type of self-harm n (%)		
Self-poisoning	214 (72)	227 (71)
Self-injury	47 (16)	53 (17)
Both	21 (7.0)	23 (7.2)
Other/unknown	16 (5.3)	15 (4.7)
Previous self-harm n (%)		
Yes	215 (72)	364 (83)
No	54 (18)	46 (15)
Unknown	29 (9.7)	8 (2.5)
Previous inpatient psych treatment n (%)		
Yes	79 (75)	71 (73)
No	22 (21)	25 (26)
Unknown	4 (3.8)	1 (1.0)
Number of people presenting	298	318
Repeat episodes within Jan/Feb/Mar	75	63
Repeat episodes in Apr/May/Jun within 90 days from index episode	*30*	*34*
Total episodes	*403*	*415*
Number of SH episodes per patient (<90 days)		
1	250	265
2	36	34
3	5	11
4	2	2
> 4	5	6
Max	19 (n = 1)	9 (n = 1)

For peer review only - http://bmjopen.bmj.com/site/about/guidelines.xhtml

Table 2 Differences in the process of care before and after the extended LPS[1]

	Jan - Mar 2014 (n = 373)	Jan- Mar 2015 (n = 381)	Difference (abs)	p-value
PS assessment n (%)[1]	213 (57)	258 (68)	+45 (+11%)	0.003
Assessor for those who had a, n (%)[1]	(n = 213)	(n = 258)		
Psychiatrist	109 (51)	77 (30)	-32 (-21%)	<0.001
Liaison Nurse	71 (33)	166 (64)	+95 (+31%)	
Other	24 (5.9)	13 (5.0)	-1 (-0.9%)	
Unknown	9 (5.9)	2 (0.8)	-10 (-5.1%)	
PS assessment by hour of day, n (%)[1]				
Monday to Friday 0900-1700	133 (62)	117 (46)	-16 (-16%)	<0.001
Other	61 (29)	121 (47)	+60 (+18%)	
Unknown	19 (8.9)	20 (7.8)	+1 (-1.1%)	
Median time from ED arrival to PS assessment[2]	11h44m	8h30m	-3h14m	<0.017
Median time from medical assessment to PS assessment[2]				
Overall	9h30min (n=185)	6h53min (n=230)	-2h37m	<0.001
Attendances between 09:00 and 17:00 hrs	2h51min (n = 44)	2h59min (n=60)	+8m	0.078
Attendances during other hours	10h20min (n=141)	8h28min (n=170)	-1h52m	0.003
Admission to ward, n (%)[1]				
No	121 (32)	117 (31)	-4 (-1%)	0.110
Yes – general ward	212 (58)	238 (63)	+26 (+5%)	
Yes – ITU	9 (2.5)	2 (0.5)	-7 (-2%)	
Yes – other	24 (6.6)	21 (5.6)	-3 (-1%)	
Median (p25-75) duration of hospital stay if not admitted to ward (hours)	No data	12 (7-21)		
Mean duration of hospital stay if admitted to ward/ITU (days) [4]	1.7 (4.1)	1.4 (2.8)	-0.37	0.26[4]
Median (p25-75) duration of hospital stay if admitted to ward/ITU (days) [5]	1 (1-1)	1 (1-1)	=	0.004
Total admission days (incl. ITU)	480	393	-87	
Outcome of ED attendance, n (%)[1]				
Psychiatric inpatient admission	11 (2.9)	11 (2.9)	-/-	0.960
Intensive mental health team	0 (0)	8 (2.1)	+8 (+2.1%)	0.008
Other community team	27 (7.2)	58 (15)	+31 (+8%)	0.001
Self-harm clinic	57 (15)	18 (4.7)	-39 (-10%)	<0.001
Alcohol nurse service	19 (5.1)	20 (5.2)	+1 (+0.1%)	.923
Home/GP care only	94 (25)	89 (23)	-5 (-2%)	0.555
Social services	0 (0)	3 (0.8)	+3 (+0.8%)	0.086
Voluntary agency	0 (0)	13 (3.4)	+13 (+3.4%)	<0.001
Custody	25 (6.7)	14 (3.7)	-11 (-3%)	0.061
Died	0 (0)	1 (0.3)	+1 (+0.3%)	0.505
Crisis service	46 (12)	50 (13)	+4 (+1%)	0.745
Patients self-discharging, n (%)[1]	73 (20)	51 (13)	-22 (-7%)	0.02
Episodes with repeat self-harm <90 days, n (%)				
Total repeat episodes (min-max)[6]	105 (0 – 19)	97 (0 – 9)	-8 (-8%)	0.79
First repeat after index, n (%)	48 (18)	54 (17)	+6 (-1%)	
- first episode of self-harm , n (%)	3 (5.6)	2 (4.3)	-1 (-1.3%)	
- previous self-harm, n (%)	45 (21)	52 (20)	+7 (-1%)	

1 p-value based on X^2 test.
2 p-value based on log-rank test.
3 p-value based on Cox-regression, adjusted for previous self-harm, sex and suicide risk.
4 p-value based on bootstrap corrected t-test statistic.
5 p-value based on Wilcoxon-Mann-Witney test.
6 p-value based on Wilcoxon-Mann-Witney test for # attendances per patient.

For peer review only - http://bmjopen.bmj.com/site/about/guidelines.xhtml

FIGURES

Figure 1 Time between arrival at ED and psychosocial assessment (log-rank test p-value: 0.001)

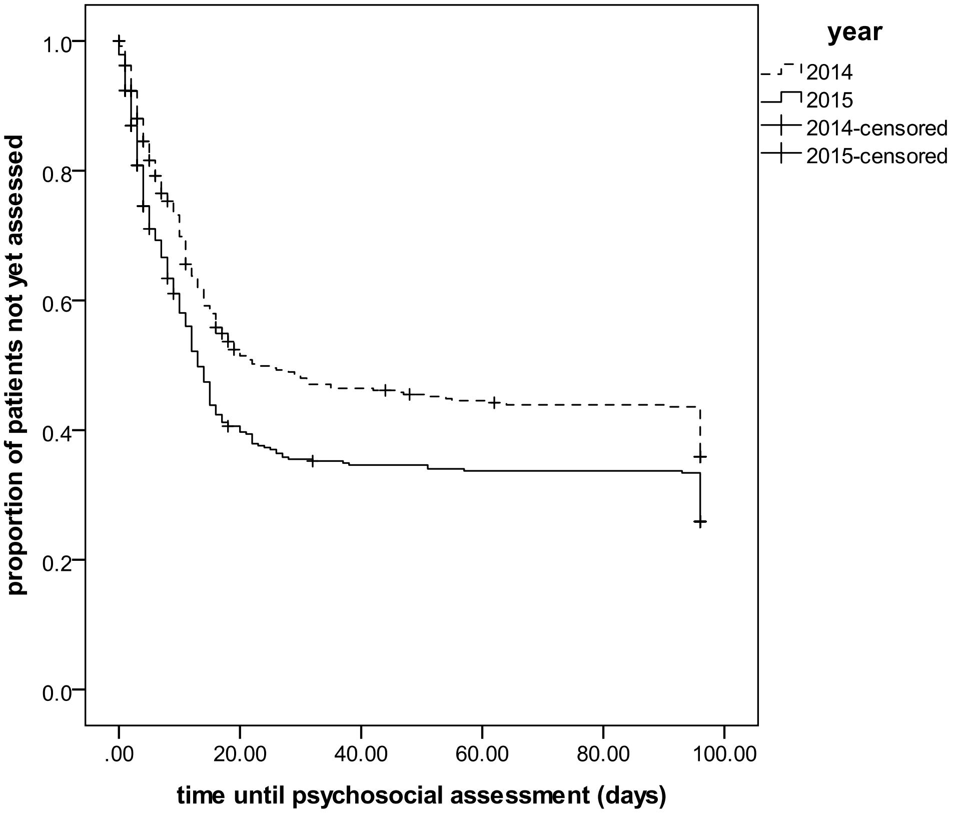

For peer review only - http://bmjopen.bmj.com/site/about/guidelines.xhtml

Figure 2 Kaplan-Meier curves comparing repeat episodes of self-harm following the index attendance in patients with No previous self-harm (A); Previous self-harm (B); and All repeat self-harm attendances < 90 days (C) for 2014 and 2015.

Repeat self-harm after index attendance

2A No previous self-harm

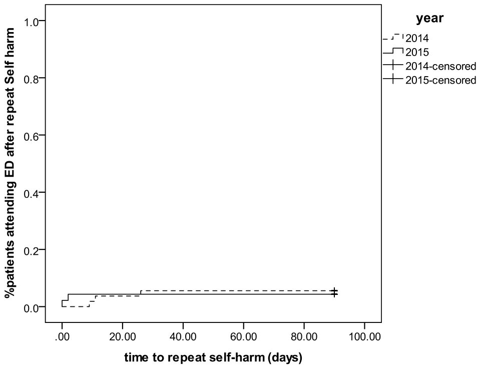

2B Patients with previous self-harm

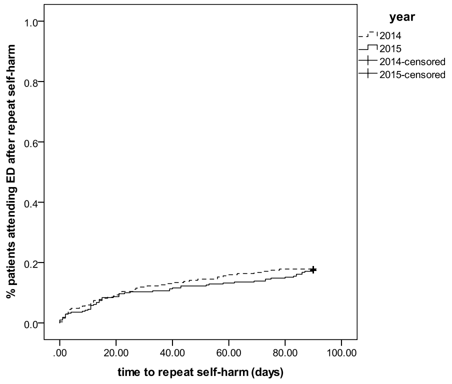

2C All episodes of repeat self-harm < 90days

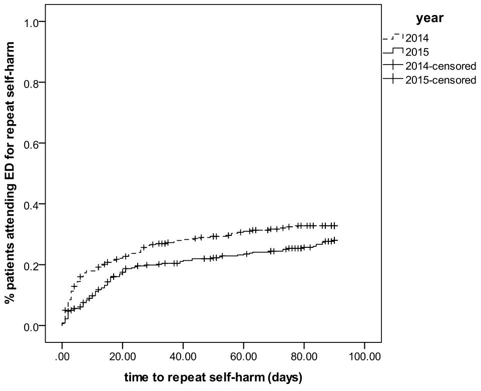

For peer review only - http://bmjopen.bmj.com/site/about/guidelines.xhtml

REFERENCES

1. Hawton K, Bergen H, Casey D, Simkin S, Palmer B, Cooper J, et al. Self-harm in England: a tale of three cities. Multicentre study of self-harm. Soc Psychiatry Psychiatr Epidemiol. 2007;42(7):513-21.
2. Cooper J, Steeg S, Bennewith O, Lowe M, Gunnell D, House A, et al. Are hospital services for self-harm getting better? An observational study examining management, service provision and temporal trends in England. BMJ Open. 2013;3(11):e003444.
3. Carroll R, Metcalfe C, Gunnell D. Hospital presenting self-harm and risk of fatal and non-fatal repetition: systematic review and meta-analysis. PLoS One. 2014;9(2):e89944.
4. Hawton K, Zahl D, Weatherall R. Suicide following deliberate self-harm: long-term follow-up of patients who presented to a general hospital. Br J Psychiatry. 2003;182:537-42.
5. Zahl DL, Hawton K. Repetition of deliberate self-harm and subsequent suicide risk: long-term follow-up study of 11,583 patients. Br J Psychiatry. 2004;185:70-5.
6. CG16 Self-harm: The short-term physical and psychological management and secondary prevention of self-harm in primary and secondary care: National Institute for Clinical Excellence (NICE); 2004 [Available from: https://www.nice.org.uk/guidance/cg16/evidence/full-guideline-189936541.
7. Kapur N, House A, Creed F, Feldman E, Friedman T, Guthrie E. Management of deliberate self poisoning in adults in four teaching hospitals: descriptive study. BMJ. 1998;316(7134):831-2.
8. Kapur N, House A, Dodgson K, May C, Creed F. Effect of general hospital management on repeat episodes of deliberate self poisoning: cohort study. BMJ. 2002;325(7369):866-7.
9. Carroll R, Metcalfe C, Steeg S, Davies NM, Cooper J, Kapur N, et al. Psychosocial Assessment of Self-Harm Patients and Risk of Repeat Presentation: An Instrumental Variable Analysis Using Time of Hospital Presentation. PLoS One. 2016;11(2):e0149713.
10. Bennewith O, Gunnell D, Peters T, Hawton K, House A. Variations in the hospital management of self harm in adults in England: observational study. BMJ. 2004;328(7448):1108-9.
11. Parsonage M, Fossey M. Economic evaluation of a liaison psychiatry service: Centre of Mental Health; 2011 [Available from: https://www.centreformentalhealth.org.uk/evaluation-liaison-psychiatry.
12. Craig P, Cooper C, Gunnell D, Haw S, Lawson K, Macintyre S, et al. Using natural experiments to evaluate population health interventions: guidance for producers and users of evidence: Medical Research Council (MRC); 2010 [Available from: https://www.mrc.ac.uk/documents/pdf/natural-experiments-guidance/.
13. Gilbody S, House A, Owens D. The early repetition of deliberate self harm. J R Coll Physicians Lond. 1997;31(2):171-2.

For peer review only - http://bmjopen.bmj.com/site/about/guidelines.xhtml

14. Fitzmaurice GM, Laird NM, Ware JH. Applied Longitudinal Analysis. Second Edition ed. Hoboken, New Jersey: John Wiley & Sons; 2011.
15. NHS reference costs 2014 to 2015: Department of Health (DH); 2015 [Available from: https://www.gov.uk/government/publications/nhs-reference-costs-2014-to-2015.
16. The mental health strategy for England - No Health Without Mental Health: a cross-government mental health outcomes strategy for people of all ages: Department of Health (DH); 2011 [Available from: https://www.gov.uk/government/uploads/system/uploads/attachment_data/file/213761/dh_124058.pdf.

For peer review only

For peer review only - http://bmjopen.bmj.com/site/about/guidelines.xhtml

Table 3 Totals per attendance for index patients in Jan-Mar 2014 and 2015 and repeated self-harm episodes within 90 days

		**2014** (n = 403 attendances*)		**2015** (n = 415 attendances*)		**difference**		
**unit**	**unit costs**	**Volume**	**Costs**	**Volume**	**Costs**	**Volume**	**Costs**	**(95%BCI**)**
**ED attendances**								
ED	£133.20	0.35	£46	0.32	£42	-0.03	-£4	
ED + admission	£205.85	0.65	£134	0.68	£140	0.03	£6	
LPS referral	£187.45	0.55	£103	0.67	£126	0.12	£23	
subtotal ED attendances			*£283*		*£309*		*£25*	*(£11 to £39)*
**BRI admissions**								
observational ward (days)	£405.05	0.97	£391	0.83	£335	-0.1	-£56	
ITU (days)	£1,058.75	0.03	£29	0.01	£13	-0	-£16	
other ward (days)	£405.05	0.2	£80	0.11	£44	-0.1	-£36	
subtotal admissions			*£500*		*£391*		*-£109*	*(-£276 to £50)*
**MEAN TOTAL COSTS PER ATTENDANCE**			**£784**		**£700**		**-£84**	**(-£254 to £77)**
TOTAL COSTS FOR COHORT			£315,843		£290,562		-£25,281	

* cost estimates are based on all attendances within 90 days after index attendance; figures may therefore differ slightly from those in Table 2

** 95% bootstrap confidence interval based on 1000 bias corrected accelerated bootstraps

For peer review only

Table 4 Sensitivity analyses: mean costs per attendance, difference between 2014 and 2015 and total cost impact for cohort for different assumptions and estimates

Analysis	*Description*	*mean cost per attendance*			*total cohort*
**nr**	**Analysis**	**2014**	**2015**	**difference**	**difference**
0	Main analysis (base-case)	£784	£700	-£84	-£25,281
1	Differentiate PS assessment by Liaison nurse /Psychiatrist	£783	£685	-£98	-£31,174
2	Ignore ITU admissions	£766	£692	-£74	-£21,328
3	Unit cost LPS referral - low (-25%)	£758	£669	-£89	-£27,997
4	Unit cost LPS referral - high (+25%)	£809	£732	-£78	-£22,565
5	Unit cost Ward admission - low (-25%)	£666	£605	-£60	-£17,076
6	Unit cost Ward admission - high (+25%)	£902	£795	-£107	-£33,486

For peer review only

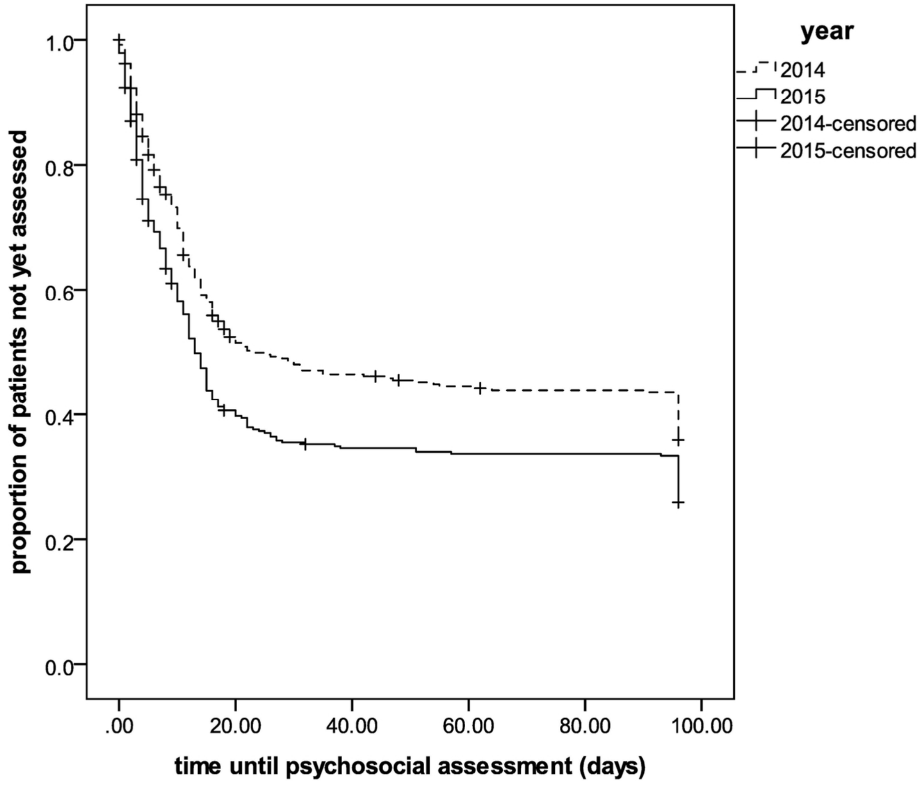

Figure 1 Time between arrival at ED and psychosocial assessment (log-rank test p-value: 0.001)

131x105mm (300 x 300 DPI)

For peer review only

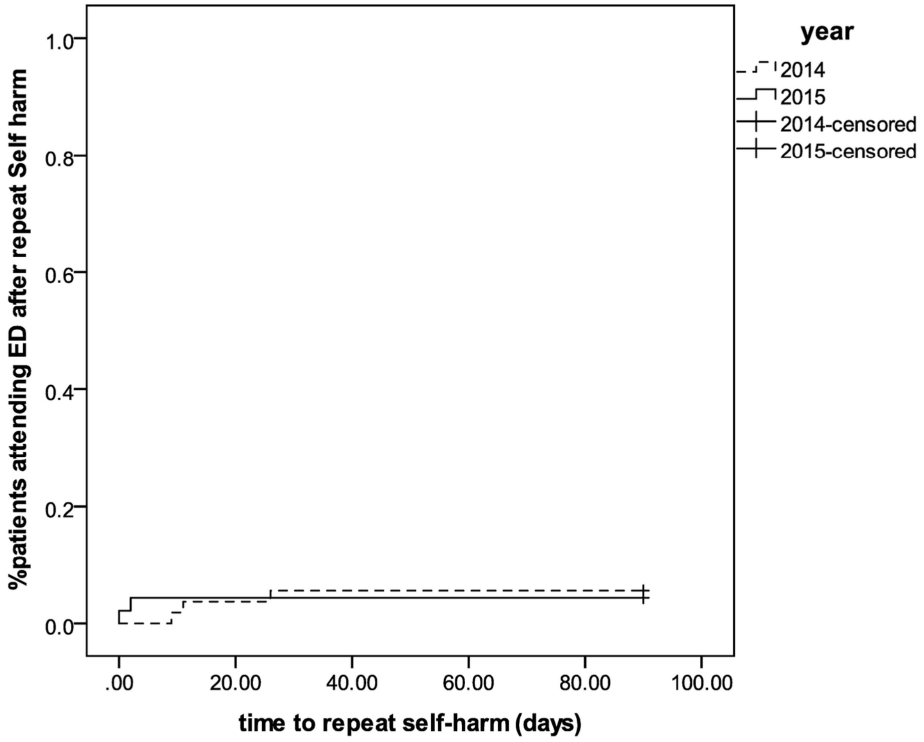

Figure 2 Kaplan-Meier curves comparing repeat episodes of self-harm following the index attendance in patients with No previous self-harm (A); Previous self-harm (B); and All repeat self-harm attendances < 90 days (C) for 2014 and 2015.
2A No previous self-harm

131x105mm (300 x 300 DPI)

only

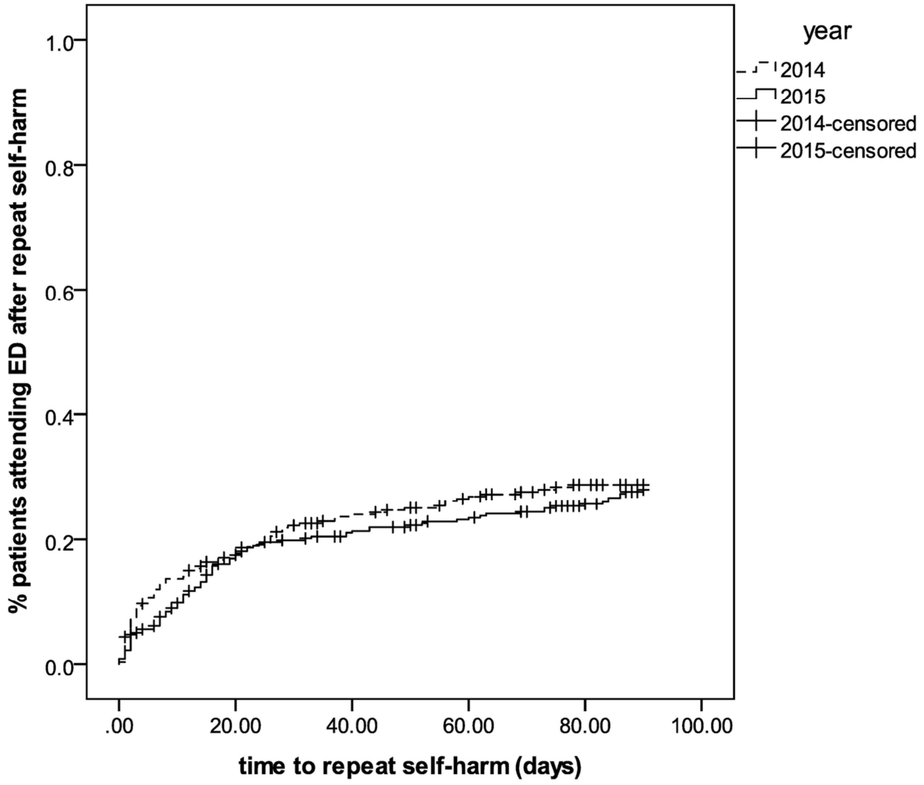

2B Patients with previous self-harm

131x105mm (300 x 300 DPI)

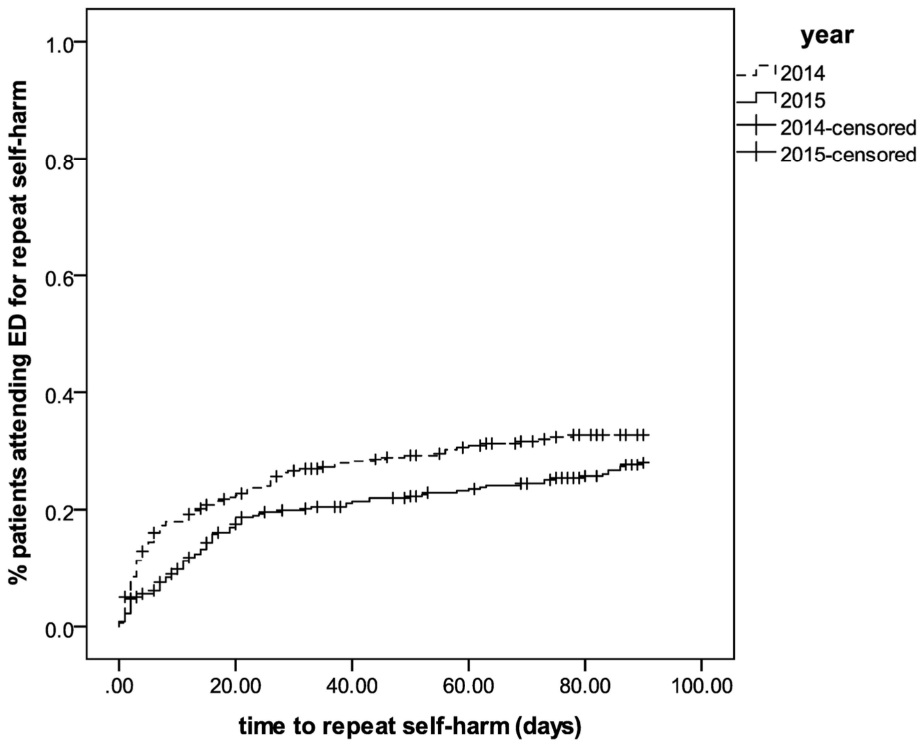

2C All episodes of repeat self-harm within 90 days

131x105mm (300 x 300 DPI)

w only

STROBE Statement—checklist of items that should be included in reports of observational studies

	Item No	Recommendation
**Title and abstract**	1	(*a*) Indicate the study's design with a commonly used term in the title or the abstract **[See page 1 (title page) and method section of the abstract page 2]**
		(*b*) Provide in the abstract an informative and balanced summary of what was done and what was found **[See results section of abstract page 2]**
**Introduction**		
Background/rationale	2	Explain the scientific background and rationale for the investigation being reported **[See Introduction page 4]**
Objectives	3	State specific objectives, including any prespecified hypotheses **[See Introduction page 5]**
**Methods**		
Study design	4	Present key elements of study design early in the paper **[See Methods page 6]**
Setting	5	Describe the setting, locations, and relevant dates, including periods of recruitment, exposure, follow-up, and data collection **[See Methods pages 6]**
Participants	6	(*a*) *Cohort study*—Give the eligibility criteria, and the sources and methods of selection of participants. Describe methods of follow-up **[See Methods page 6]** *Case-control study*—Give the eligibility criteria, and the sources and methods of case ascertainment and control selection. Give the rationale for the choice of cases and controls *Cross-sectional study*—Give the eligibility criteria, and the sources and methods of selection of participants
		(*b*) *Cohort study*—For matched studies, give matching criteria and number of exposed and unexposed *Case-control study*—For matched studies, give matching criteria and the number of controls per case
Variables	7	Clearly define all outcomes, exposures, predictors, potential confounders, and effect modifiers. Give diagnostic criteria, if applicable **[See Methods page 6 -7]**
Data sources/ measurement	8*	For each variable of interest, give sources of data and details of methods of assessment (measurement). Describe comparability of assessment methods if there is more than one group **[See Methods page 6 -7]**
Bias	9	Describe any efforts to address potential sources of bias **[See Methods page 6 (participants and data) and page 7 (statistical analysis)]**
Study size	10	Explain how the study size was arrived at **[See Methods page 6]**
Quantitative variables	11	Explain how quantitative variables were handled in the analyses. If applicable, describe which groupings were chosen and why **[See Methods page 7]**
Statistical methods	12	(*a*) Describe all statistical methods, including those used to control for confounding **[See Methods page 7]**
		(*b*) Describe any methods used to examine subgroups and interactions **[See Methods page 7]**
		(*c*) Explain how missing data were addressed **[See Methods page 6]**
		(*d*) *Cohort study*—If applicable, explain how loss to follow-up was addressed **[N/A]** *Case-control study*—If applicable, explain how matching of cases and controls was addressed *Cross-sectional study*—If applicable, describe analytical methods taking account of

For peer review only

		sampling strategy
		(*e*) Describe any sensitivity analyses **[See Methods page 8]**
**Results**		
Participants	13*	(a) Report numbers of individuals at each stage of study—eg numbers potentially eligible, examined for eligibility, confirmed eligible, included in the study, completing follow-up, and analysed **[See page 9;table 1]**
		(b) Give reasons for non-participation at each stage **[N/A]**
		(c) Consider use of a flow diagram **[Information presented in table 1]**
Descriptive data	14*	(a) Give characteristics of study participants (eg demographic, clinical, social) and information on exposures and potential confounders **[See page 9;table 1]**
		(b) Indicate number of participants with missing data for each variable of interest **[See table 1]**
		(c) *Cohort study*—Summarise follow-up time (eg, average and total amount) **[N/A]**
Outcome data	15*	*Cohort study*—Report numbers of outcome events or summary measures over time **[See pages 9-10;table 2;figures 1 and 2]**
		Case-control study—Report numbers in each exposure category, or summary measures of exposure
		Cross-sectional study—Report numbers of outcome events or summary measures
Main results	16	(*a*) Give unadjusted estimates and, if applicable, confounder-adjusted estimates and their precision (eg, 95% confidence interval). Make clear which confounders were adjusted for and why they were included **[See pages 9-10;table 2;figures 1 and 2]**
		(*b*) Report category boundaries when continuous variables were categorized **[N/A]**
		(*c*) If relevant, consider translating estimates of relative risk into absolute risk for a meaningful time period **[N/A]**
Other analyses	17	Report other analyses done—eg analyses of subgroups and interactions, and sensitivity analyses **[See pages 9-10;tables 3 and 4;figure 2]**
**Discussion**		
Key results	18	Summarise key results with reference to study objectives **[See page 11]**
Limitations	19	Discuss limitations of the study, taking into account sources of potential bias or imprecision. Discuss both direction and magnitude of any potential bias **[See page 11]**
Interpretation	20	Give a cautious overall interpretation of results considering objectives, limitations, multiplicity of analyses, results from similar studies, and other relevant evidence **[See pages 11-12]**
Generalisability	21	Discuss the generalisability (external validity) of the study results **[See page 11]**
**Other information**		
Funding	22	Give the source of funding and the role of the funders for the present study and, if applicable, for the original study on which the present article is based **[See pages 13]**

*Give information separately for cases and controls in case-control studies and, if applicable, for exposed and unexposed groups in cohort and cross-sectional studies.

Note: An Explanation and Elaboration article discusses each checklist item and gives methodological background and published examples of transparent reporting. The STROBE checklist is best used in conjunction with this article (freely available on the Web sites of PLoS Medicine at http://www.plosmedicine.org/, Annals of Internal Medicine at http://www.annals.org/, and Epidemiology at http://www.epidem.com/). Information on the STROBE Initiative is available at www.strobe-statement.org.

For peer review only - http://bmjopen.bmj.com/site/about/guidelines.xhtml

Extending the liaison psychiatry service in a large hospital in the UK: a before and after evaluation of the economic impact and patient care following Emergency Department attendances for self-harm

Journal:	*BMJ Open*
Manuscript ID	bmjopen-2017-016906.R1
Article Type:	Research
Date Submitted by the Author:	05-Jun-2017
Complete List of Authors:	Opmeer, Brent; NIHR Collaboration for Leadership in Applied Health Research and Care West; AMC, Clinical Research Unit<br>Hollingworth, William; University of Bristol, School of Social and Community Medicine<br>Marques, Elsa; University of Bristol, School of Clinical Sciences<br>Margelyte, Ruta; University of Bristol, School of Social and Community Medicine; NIHR Collaboration for Leadership in Applied Health Research and Care West,<br>Gunnell, David; Bristol University, School of Social and Community Medicine
**Primary Subject Heading**:	Health services research
Secondary Subject Heading:	Health economics, Mental health
Keywords:	Suicide & self-harm < PSYCHIATRY, psychosocial assesement, service evaluation, ACCIDENT & EMERGENCY MEDICINE, liaison psychiatry

SCHOLARONE™ Manuscripts

Title

Extending the liaison psychiatry service in a large hospital in the UK: a before and after evaluation of the economic impact and patient care following Emergency Department attendances for self-harm

Authors

B.C. Opmeer[1,2], W. Hollingworth[1,3], E. Marques[4] , R. Margelyte[1,3], D. Gunnell[2]

Author affiliations:

[1] The National Institute for Health Research Collaboration for Leadership in Applied Health Research and Care (CLAHRC) West at University Hospitals Bristol
[2] Clinical Research Unit, Academic Medical Centre, Amsterdam, the Netherlands
[3] School of Social and Community Medicine, University of Bristol, UK
NHS Foundation Trust, Bristol, UK
4 School of Clinical Sciences, University of Bristol, UK

Corresponding author

Dr Brent Opmeer, NIHR CLAHRC West at University Hospitals Bristol NHS Foundation Trust, 9th Floor, Whitefriars, Lewins Mead, Bristol BS1 2NT, UK.
bo15082@bristol.ac.uk, phone: +31-624 548 900

For peer review only

For peer review only - http://bmjopen.bmj.com/site/about/guidelines.xhtml

ABSTRACT

Objectives: To evaluate the impact of an expansion of Liaison Psychiatry Services (LPS) on patient management, outcomes and treatment costs for Emergency Department (ED) attendances for self-harm.

Design: Retrospective before and after cohort study using routinely collected Self-Harm Surveillance Register data.

Setting: A large hospital in South West England.

Subjects: Patients attending the ED for self-harm.

Interventions: Extension of the Liaison Psychiatry Service's (LPS) working hours from 9am to 5pm Monday to Friday to 8am to 10pm 7 days a week, following a £250,000 annual investment

Main outcome measures: Number and characteristics of ED attendances for self-harm. The before and after cohorts were compared in terms of key process measures, including proportion of patients receiving a psychosocial assessment, average length of hospital stay, waiting times for assessment, proportion of patients who self-discharged without an assessment, levels of repeat self-harm attendances and mean cost per patient attendance.

Results: 298 patients attended ED for self-harm on 373 occasions between January and March 2014, and 318 patients attended on 381 occasions between January and March 2015. The proportion of ED attendances where patients received a psychosocial assessment increased from 57% to 68% (p=0.003), median waiting time decreased by 3h14min (p=0.017), and the proportion of episodes where patients self-discharged without a psychosocial assessment decreased from 20% to 13% (p=0.022). The mean cost per patient attendance was marginally lower after the intervention (-£84; 95%CI: -£254 to £77).

Conclusions: The extended LPS seems to have had a favourable effect on the management and outcomes of self-harm patients. The cost of extending the LPS's working hours might be partially offset by more efficient assessment and discharge. The impact of the extended LPS on the care of hospitalised patients with mental health problems other than self-harm requires further evaluation.

For peer review only - http://bmjopen.bmj.com/site/about/guidelines.xhtml

SUMMARY

Strengths:

- There was a major step-change in care provision (increase in service availability from 40 to 98 hours per week), providing a good opportunity to evaluate the impact of changed service provision

- Detailed and relatively complete individual level patient data were available from a bespoke self-harm register, facilitating estimation of resource costs for self-harm patients.

Limitations:

- Analysis does not assess the wider impact of the extended LPS on post-discharge service provision, and on patients with other mental health conditions seen by the LPS.

- Analysis does not include a control site for wider control of economic, social and political trends in mental health and service provision.

- The sample size was relatively small and we lacked the power to detect important impacts on hospital costs.

For peer review only

For peer review only - http://bmjopen.bmj.com/site/about/guidelines.xhtml

INTRODUCTION

There are an estimated 200,000 Emergency Department (ED) attendances for self-harm in England & Wales every year(1) ; approximately half of these result in admission to a hospital ward(2). Self-harm is often repeated, with more than 15% of individuals who attend a hospital with self-harm re-attending within a year(3). A history of self-harm is the strongest risk factor for suicide across a range of psychiatric disorders(4). Repeated self-harm further increases suicide risk(5). Providing effective, evidence based clinical care for this high-risk patient population is a key means of reducing their risk of subsequent self-harm and suicide.

UK clinical guidelines suggest that all patients should be offered a psychosocial assessment after self-harm. This should include an evaluation of the factors leading to self-harm and suicidal intent together with a full mental health and social needs assessment(6). However in many UK hospitals, more than half of patients are discharged from the emergency department without an assessment(2). Patients who leave the ED without a psychosocial assessment are less likely to be offered follow-up(7). There is also evidence that psychosocial assessments reduce risk of repeat self-harm(8, 9).

Liaison Psychiatry Services (LPS) have been introduced in hospitals to provide assessment and care for patients presenting to the ED with mental health problems and to support people with physical health problems who also have, or develop, mental health problems such as delirium whilst they are in hospital. Despite the existing guidelines on the management of self-harm(6), there are significant variations in service models in terms of staffing, coverage or management(2, 10). Most people who have self-harmed seek help at times when only an emergency mental health service is available. One response is to invest in extended LPS operating hours, but the benefits of such investment have not been well established. An exception is the economic evaluation of expanded LPS service at a large acute hospital in Birmingham which found that additional investment in the services generated incremental benefits in terms of reduced bed use with overall benefit to cost ratio of more than 4:1 (11).

In 2014, a local Clinical Commissioning Group (CCG) invested approximately £250,000 per annum in an extended LPS at a large teaching hospital with a consultant-led 24-hour ED. This was used to increase the working hours of the LPS from Monday to Friday 0900-1700 (i.e. 40 hours per week) to seven days a week 0800-2200 (98 hours per week). To achieve this increase in service provision, four additional full time liaison nurses were employed. The aim was to increase the proportion of patients attending the emergency department after self-harm who receive a psychosocial assessment and reduce admissions to acute hospital beds to await LPS assessment. It was anticipated that these changes might also lead to better patient outcomes such as reduced repeat self-harm and suicide as a result of increases in the proportion of patients receiving appropriate follow-up care.

For peer review only - http://bmjopen.bmj.com/site/about/guidelines.xhtml

Our primary objective was to assess the impact of the extended LPS on process measures and indicators of patient outcomes and costs following self-harm. We assessed resource use and costs associated with the management of patients attending the emergency department following self-harm. Our findings will enable commissioners to explore whether extra investment in LPS can improve patient management and outcomes of self-harm and result in cost savings.

For peer review only

For peer review only - http://bmjopen.bmj.com/site/about/guidelines.xhtml

METHODS

Study design

The change in LPS provision is a natural experiment(12) as there has been a step-change in the availability of a service from a defined point in time (1st of September 2014). We compared the process, cost and outcomes of care for patients attending the ED following self-harm in a before and after study. We estimated NHS secondary care costs, although we recognise that care provided by the LPS will have spill-over effects on primary and community care services.

Participants and data

We compared two patient cohorts. The first consisted of patients presenting to the ED following self-harm before the operating hours of LPS were extended (January 1st – March 31st 2014). The second consisted of patients presenting after the extended LPS was fully operational (January 1st – March 31st 2015). Our focus was on self-harm patients because these are the group of psychiatric patients most likely to be admitted to a hospital bed, and they were the focus of the new funding. However, it is important to note that self-harm patients comprise only 40% of the LPS workload, therefore the extended service will have an impact on a wider group of patients.

Patients were identified from the local Self-Harm Surveillance Register (SHSR). The SHSR was established in 2010 and records clinical and sociodemographic details of all hospital-presentations for self-harm at the hospital. Using the SHSR we identified the index self-harm ED attendances in each period (i.e. the first time a patient attended between January 1st and March 31st) and any repeat self-harm ED attendance within 90 days of the index episode. The follow-up period was defined on the basis that a high proportion of all patients who repeat self-harm within 12 months do so within 90 days of the index event(13).

We selected time periods which are not adjacent to the service change date (September 2014) to avoid the period when the service might have been 'ramping up' or 'bedding in'. We selected the same three calendar months for the before and after periods to avoid bias due to seasonal trends in mental health and self-harm.

Each episode of self-harm was characterised in terms of patient age, sex, employment status, marital status, method of self-harm, previous self-harm or inpatient psychiatric care and a matrix risk assessment. The matrix is a locally developed tool for use by ED staff to determine the urgency with which a patient should be referred for psychosocial assessment. Patients are categorised into three groups (red, amber or green) depending on the degree of urgency. These levels guide clinical staff in deciding whether a patient should be referred for an immediate psychosocial assessment .

We assessed the following measures of care: whether a patient received a psychosocial assessment; the profession of the person carrying out the assessment; waiting times from attendance to assessment; proportion of

For peer review only - http://bmjopen.bmj.com/site/about/guidelines.xhtml

patients self-discharging from the ED without assessment; proportion of episodes admitted to a hospital ward; referrals made to other agencies / health teams; and length of hospital stay.

The patient outcome measures we evaluated were: the proportion of patients with repeat ED attendances for self-harm; number of repeat self-harm ED attendances; and time to repeated self-harm attendance. We also evaluated mean cost per self-harm ED episode and mean cost per patient (including repeat self-harm episodes within 90 days).

Data collection is approved by the Central Bristol Research Ethics Committee.

Data quality/missing data

Audits reveal SHSR case ascertainment is >95%. Data extracted from the SHSR were checked for inconsistencies, and where evident these were resolved.
The two main types of inconsistency that were identified were date errors (e.g. dates of discharge preceded data of attendance) and variables reflecting composite questions, where the first variable was missing (e.g. admitted to ward), but the second variable was completed (e.g. date of admission to ward). In these evident cases, inconsistent or missing values were corrected with consistent values.
Multiple imputation (SAS Proc MI) was used, with 15 imputation rounds, to avoid exclusion of observations due to missing data in multivariable analyses (see below). Estimates of effects and standard errors from analyses based on each imputed dataset were subsequently pooled (SAS Proc MIanalyze), reflecting the uncertainty due to the imputation of missing values.

Statistical analyses

Analyses of patient characteristics were based on index attendances during the three-month periods (January-March) in 2014 and 2015. Analyses of the impact on service delivery are based on all attendances (including repeat presentations) in each three month period (in 2014 and 2015). Analyses describing the impact on risk of repeat self-harm are based on index attendances and all subsequent attendances for repeat self-harm within 90 days associated with these index attendances (i.e. including attendances up to June).

Descriptive analyses

Characteristics of the study population in each three month period are reported descriptively. Continuous variables are summarised as medians, means and standard deviations as appropriate, for categorical variables, the number and percentage of participants/attendances within each category are presented.

Evaluation of impact

Differences in the process of care are examined for attendances before and after the extended LPS became operational. Proportions, means and medians for service outcome measures by year are reported with the absolute difference between the years and associated p-values (two tailed chi-square or t-test). This includes the proportion of attendances that received a psychosocial assessment

For peer review only - http://bmjopen.bmj.com/site/about/guidelines.xhtml

within or outside LPS service hours, the professional background of the assessor, the median times from ED arrival or medical assessment to psychosocial assessment, the proportion of attendances admitted to an observation ward, general ward or intensive therapy unit (ITU) and the median duration of hospital stay after admission. As the length of ITU care for patients admitted to the ITU was not documented, we assumed this duration to be 30% of the total stay, with a minimum of 1 day, for these patients. The proportion of patients that self-discharged and the number of episodes of repeated self-harm within 90 days are also considered as potential outcomes of the LPS activities.

Kaplan Meier analyses were used to compare differences in time between ED arrival and psychosocial assessment before and after extended LPS.

Differences in time until repeat self-harm attendances within 90 days were also compared for 2014 and 2015 using Kaplan-Meier analyses and Cox proportional hazards regression to adjust for relevant factors (previous self-harm, age, sex, and Matrix risk) associated with re-attendance rates. Time to repeat self-harm was compared before and after the extended LPS for all patients and for subgroups of patients with and without previous self-harm. We used SAS proc PHREG, a proportional hazards regression analysis that allowed for multiple repeat self-harm episodes during the 90 days follow-up, and used the robust “sandwich” estimator to account for correlated observations within the same patient (i.e. that some patients are more likely to repeat self-harm than others)(14).

Economic analysis

Mean cost per attendance was estimated for patients presenting in the periods before and after the LPS was extended. This analysis was based on index presentation and all repeat attendances during the 90 days follow-up. All unit costs were estimated from the 2014/15 NHS reference costs(15). NHS reference costs for ED care are higher for patients subsequently admitted to a hospital bed (mean = £205.85) than for patients who are discharged from the ED (mean = £133.20). The NHS reference cost for an emergency department mental health liaison contact (£187) does not distinguish between those conducted by psychiatrists or nursing staff. We used this figure in our analysis, but the actual cost may be lower for assessments conducted by nurses. The cost of inpatient care depends on the ward type, the type of treatment required and the length of stay. Any reductions in hospital admissions due to the extended LPS may be due to fewer short stay admissions to observation units or other wards while waiting for psychosocial assessment. Therefore, we used the average unit cost (£405.50) for a non-elective short stay admission for 'observation or counselling' as a proxy for the per diem cost of observational unit or other ward care. We used the average daily cost (£1,058.75) of adult medical critical care patients to estimate costs for ITU days. In view of the skewed distribution of health care costs, the SPSS bootstrap procedure was used to estimate 95% confidence intervals for cost estimates, based on 1000 replications.

For peer review only - http://bmjopen.bmj.com/site/about/guidelines.xhtml

Robustness of the findings was assessed in the following univariate sensitivity analyses. We increased/decreased unit cost estimates for ward admissions as well as for LPS assessments by 25%. We differentiated between psychosocial assessment carried out by a liaison nurse or a psychiatrist by decreasing nurse costs and increasing psychiatrist costs by 25% each. Finally, we applied observational ward costs for all hospital days including ITU days, on the assumption that LPS may reduce hospital days but is very unlikely to have any effect on ITU days.

Data preparation, tables, figures and analyses are documented and performed using statistical software SPSS version 23, and SAS version 9.4.

For peer review only

For peer review only - http://bmjopen.bmj.com/site/about/guidelines.xhtml

RESULTS

Similar numbers of patients attended the ED following self-harm between January and March 2014 (n=298) and January and March 2015 (n=318) (Table 1). Only around 20% of ED attendances in 2014 and 2015 occurred during the original LPS working hours (Mon-Fri 0900-1700).

Overall, details in patient characteristics and patient care were accurately documented in the self-harm register. The number (%) of people with missing data are reported in Tables 1 and 2. For key variables, e.g. whether an assessment was performed, previous self-harm, time of assessment, time of discharge, outcome of ED attendance, completeness ranged from 0% (assessment performed/ outcome of attendance) to 9.7% (previous self-harm).

There are generally only minor differences in the characteristics of the self-harm patients in the two time periods. In 2015 a higher proportion of women attended following self-harm (63% vs 57%), and fewer patients were unemployed (46% vs 57%). Slightly more patients were known to have a history of self-harm (83% vs 72%), but previous self-harm was also better documented in 2015 (2.5% unknown) as compared to 2014 (9.7% unknown).

There were 105 episodes of repeat self-harm within 90 days of the index attendance in 2014, versus 97 episodes within the same timeframe in 2015 (Table 2). Including repeat episodes, the total number of ED attendances associated with index admissions in the first three months was 373 in 2014 and 381 in 2015. The average number of repeat episodes within 90 days relative to the index attendances decreased from 0.35 (105/298) in 2014 to 0.31 (97/318) in 2015.

For peer review only - http://bmjopen.bmj.com/site/about/guidelines.xhtml

Table 1 Number and characteristics of patients index episodes of ED SH attendances in 2014 and 2015 periods*

	Jan - Mar 2014 (n=298)	Jan - Mar 2015 (n = 318)
Attendances by hour of day n (%)		
Monday to Friday 0900-1700	70 (21)	75 (24)
Other	228 (79)	242 (76)
Female n (%)	166 (57)	201 (63)
Age on years mean (SD)	34 (14)	35 (15)
Marital status n (%)		
Single	220 (74)	253 (80)
Married	30 (10)	32 (10)
Other	38 (13)	26 (8)
Unknown	10 (3)	7 (2)
Occupational status n (%)		
Employed	56 (19)	56 (18)
Unemployed	171 (57)	147 (46)
Other	55 (17)	80(25)
Unknown	14 (5)	35 (11)
Type of self-harm n (%)		
Self-poisoning	214 (72)	227 (71)
Self-injury	47 (16)	53 (17)
Both	21 (7.0)	23 (7.2)
Other/unknown	16 (5.3)	15 (4.7)
Previous self-harm n (%)		
Yes	215 (72)	364 (83)
No	54 (18)	46 (15)
Unknown	29 (9.7)	8 (2.5)
Previous inpatient psych treatment n (%)		
Yes	79 (75)	71 (73)
No	22 (21)	25 (26)
Unknown	4 (3.8)	1 (1.0)
Number of people presenting	298	318
Repeat episodes within Jan/Feb/Mar	75	63
Repeat episodes in Apr/May/Jun within 90 days from index episode	*30*	*34*
Total episodes	*403*	*415*
Number of SH episodes per patient (<90 days)		
1	250	265
2	36	34
3	5	11
4	2	2
> 4	5	6
Max	19 (n = 1)	9 (n = 1)

ED: Emergency Department; SH: self-harm; SD: standard deviation

For peer review only - http://bmjopen.bmj.com/site/about/guidelines.xhtml

With extended service hours in the LPS in 2015, the proportion of patients receiving a psychosocial assessment increased (from 57% to 68%; p=0.003; Table 2). The proportion of patients receiving a psychosocial assessment outside 2014 LPS working hours increased from 29% in 2014 to 47% in 2015; p<0.001), and the median time between arrival at the ED and psychosocial assessment decreased by more than three hours (from 11h44m to 8h30m; p=0.017;Figure 1). The median time between medical assessment and psychosocial assessment decreased by two and a half hours (from 9h30m to 6h53m), and this was also evident in the subgroup of patients attending during the original LPS office hours (from 10h20m to 8h28min; p=0.003). The proportion of episodes where patients were admitted to a ward slightly increased (from 68% to 69%); relatively more were admitted to an observation ward (from 58% to 63%), and less often to an ITU (from 2.5% to 0.5%). The median length of stay for patients admitted to a ward remained unchanged (1 days), but the average stay decreased somewhat from 1.7 (SD 4.1) days to 1.4 (SD 2.8) days, but statistical evidence for this difference was weak (p=0.26). The number of patients self-discharging before assessment and/or follow-up arrangements decreased (from 20% to 13%; p=0.022). In 2015 patients were more often referred to the Crisis team and other community teams (increasing from 12.3% to 15.2%, and from 7.2% to 15.2% respectively), and less often to the self-harm clinic (decreasing from 15.3% to 4.7%).

<<INSERT FIGURE 1 APPROX HERE>>

For peer review only - http://bmjopen.bmj.com/site/about/guidelines.xhtml

Table 2 Differences in the process of care before and after the extended LPS

	Jan - Mar 2014 (n = 373)	Jan- Mar 2015 (n = 381)	Difference (abs)	p-value
Psychosocial assessment n (%)[1]	213 (57)	258 (68)	+45 (+11%)	0.003
Assessor for those who had a, n (%)[1]	(n = 213)	(n = 258)		
Psychiatrist	109 (51)	77 (30)	-32 (-21%)	<0.001
Liaison Nurse	71 (33)	166 (64)	+95 (+31%)	
Other	24 (5.9)	13 (5.0)	-1 (-0.9%)	
Unknown	9 (5.9)	2 (0.8)	-10 (-5.1%)	
Psychosocial assessment by hour of day, n (%)[1]				
Monday to Friday 0900-1700	133 (62)	117 (46)	-16 (-16%)	<0.001
All other times	61 (29)	121 (47)	+60 (+18%)	
Unknown	19 (8.9)	20 (7.8)	+1 (-1.1%)	
Median time from ED arrival to Psychosocial assessment[2]	11h44m	8h30m	-3h14m	<0.017
Median time from medical assessment to PS assessment[2]				
Overall	9h30min (n=185)	6h53min (n=230)	-2h37m	<0.001
Attendances between 09:00 and 17:00 hrs	2h51min (n = 44)	2h59min (n=60)	+8m	0.078
Attendances during other hours	10h20min (n=141)	8h28min (n=170)	-1h52m	0.003
Admission to ward, n (%)[1]				
No	121 (32)	117 (31)	-4 (-1%)	0.110
Yes – observation ward	212 (58)	238 (63)	+26 (+5%)	
Yes – ITU	9 (2.5)	2 (0.5)	-7 (-2%)	
Yes – other	24 (6.6)	21 (5.6)	-3 (-1%)	
Median (p25-75) duration of hospital stay if not admitted (hours)	No data[7]	12 (7-21)		
Mean duration of hospital stay if admitted (days) [4]	1.7 (4.1)	1.4 (2.8)	-0.37	0.26[4]
Median (p25-75) duration of hospital stay if admitted to ward/ITU (days) [5]	1 (1-1)	1 (1-1)	=	0.004
Total admission days (incl. ITU)	480	393	-87	
Outcome of ED attendance, n (%)[1]				
Psychiatric inpatient admission	11 (2.9)	11 (2.9)	-/-	0.96
Crisis team	46 (12)	58 (15)	+12 (+3%)	0.25
Other community team	27 (7.2)	58 (15)	+31 (+8%)	0.001
Self-harm clinic	57 (15)	18 (4.7)	-39 (-10%)	<0.001
Alcohol nurse service	19 (5.1)	20 (5.2)	+1 (+0.1%)	0.92
Home/GP care only	94 (25)	89 (23)	-5 (-2%)	0.56
Social services	0 (0)	3 (0.8)	+3 (+0.8%)	0.086
Voluntary agency	0 (0)	13 (3.4)	+13 (+3.4%)	<0.001
Custody	25 (6.7)	14 (3.7)	-11 (-3%)	0.061
Died	0 (0)	1 (0.3)	+1 (+0.3%)	0.51
Patients self-discharging before a psychosocial assessment is carried out, n (%)[1]	73 (20)	51 (13)	-22 (-7%)	0.022
Episodes with repeat self-harm <90 days, n (%)				
Total repeat episodes (min-max)[6]	105 (0 – 19)	97 (0 – 9)	-8 (-8%)	0.79
First repeat after index, n (%)	48 (18)	54 (17)	+6 (-1%)	
- first episode of self-harm, n (%)	3 (5.6)	2 (4.3)	-1 (-1.3%)	
- previous self-harm, n (%)	45 (21)	52 (20)	+7 (-1%)	

1 p-value based on X^2 test.
2 p-value based on log-rank test.
3 p-value based on Cox-regression, adjusted for previous self-harm, sex and suicide risk.
4 p-value based on bootstrap corrected t-test statistic.
5 p-value based on Wilcoxon-Mann-Witney test.
6 p-value based on Wilcoxon-Mann-Witney test for # attendances per patient.
7 this element was not documented from the start of the self-harm register.

ITU: Intensive Treatment Unit; ED: Emergency Department; LPS: Liaison Psychiatric Service

For peer review only - http://bmjopen.bmj.com/site/about/guidelines.xhtml

There was no evidence of a reduction in repeat self-harm episodes following the introduction of extended LPS working hours either amongst patients with or without a previous self-harm episode (Figures 2A and 2B). The Kaplan-Meier analysis indicated that there was some evidence of a reduction in the incidence of repeat self-harm within 90 days of the index episode in 2015 compared to 2014. Proportional hazards regression suggested that in 2015, patients were less likely to re-attend the ED for self-harm within 90 days than those in the same period in 2014 (crude risk ratio 0.86; 95%CI: 0.51 to 1.44; Figure 2C) although statistical evidence for a difference was weak (p = 0.56). In a model controlling for patient characteristics (previous self-harm, age, sex, and matrix risk), this association appeared slightly stronger (adjusted risk ratio 0.79; 95%CI: 0.47 to 1.33; p = 0.37).

<<INSERT FIGURE 2A-C APPROX HERE>>

The average cost per attendance decreased from £784 in 2014 to £700 in 2015)), a cost reduction of -11% per episode. However, the 95% CI around the mean difference was large (mean difference -£84; 95% BCI: -£254 to £77). The higher costs of more LPS assessments may be offset by reduced costs of ITU and ward bed days (Table 3).

For peer review only - http://bmjopen.bmj.com/site/about/guidelines.xhtml

Table 3 Mean total costs per attendance for index patients in Jan-Mar 2014 and 2015 and repeated self-harm episodes within 90 days

		2014 (n = 403 attendances*)		2015 (n = 415 attendances*)		difference		
**unit**	**unit costs**	**Volume**	**Costs**	**Volume**	**Costs**	**Volume**	**Costs**	**(95%BCI**)**
**ED attendances**								
ED	£133.20	0.35	£46	0.32	£42	-0.03	-£4	
ED + admission	£205.85	0.65	£134	0.68	£140	0.03	£6	
LPS assessment	£187.45	0.55	£103	0.67	£126	0.12	£23	
subtotal ED attendances			*£283*		*£309*		*£25*	*(£11 to £39)*
**Hospital admissions**								
Observation ward (days)	£405.05	0.97	£391	0.83	£335	-0.1	-£56	
ITU (days)	£1,058.75	0.03	£29	0.01	£13	-0.02	-£16	
other ward (days)	£405.05	0.20	£80	0.11	£44	-0.09	-£36	
subtotal admissions			*£500*		*£391*		*-£109*	*(-£276 to £50)*
**MEAN TOTAL COSTS PER ATTENDANCE**			**£784**		**£700**		**-£84**	**(-£254 to £77)**
TOTAL COSTS			£315,843		£290,562		-£25,281	

* cost estimates are based on all attendances within 90 days after index attendance; figures may therefore differ slightly from those in Table 2
** 95% bootstrap confidence interval based on 1000 bias corrected accelerated bootstraps
ITU: Intensive Treatment Unit; ED: Emergency Department; LPS: Liaison Psychiatric Service

For peer review only

The average cost per patient (including repeat attendances within 90 days) decreased from £1060 in 2014 to £914 in 2015 (mean difference -£146; 95% BCI: -£433 to £138), a cost reduction of -14% per patient. The total costs for 298 patients attending the ED in the first three months of 2014 amounted to £315,843, whereas in the same period in 2015 the total costs for 318 patients attending the ED amounted to £290,562. Although more patients presented at the ED in 2015, the total costs associated with these self-harm episodes decreased by £25,281 (-8.0%). If extrapolated to a full year, this equates to savings of approximately £101,000 or £144,600 if the total is estimated excluding LPS assessment costs. This suggests that the annual investment (£250,000) in extending the LPS was associated with a cost reduction of £144,600 in the non-LPS hospital costs of care for patients presenting at the ED following self-harm despite the small increase in patient numbers.

Sensitivity analyses showed that the estimated cost per patient attending the ED was consistently lower in 2015 as compared to 2014, ranging from -£60 to -£107 (table 4). The impact on total costs amounted to savings between £68,300 and £133,900 per year.

Table 4 Sensitivity analyses: mean costs per attendance, difference between 2014 and 2015 and total cost impact for cohort for different assumptions and estimates

analysis		*mean cost per attendance*			*total cohort*
**id**	**description**	**2014**	**2015**	**difference**	**difference**
0	Main analysis (base-case)	£784	£700	-£84	-£25,281
1	Differentiate PS assessment by Liaison nurse /Psychiatrist	£783	£685	-£98	-£31,174
2	Assume observational unit costs for all bed days	£766	£692	-£74	-£21,328
3	Unit cost LPS assessment - low (-25%)	£758	£669	-£89	-£27,997
4	Unit cost LPS assessment - high (+25%)	£809	£732	-£78	-£22,565
5	Unit cost observational ward - low (-25%)	£666	£605	-£60	-£17,076
6	Unit cost observational ward - high (+25%)	£902	£795	-£107	-£33,486

PS; psychosocial; ITU: Intensive Treatment Unit; LPS: Liaison Psychiatric Service

For peer review only - http://bmjopen.bmj.com/site/about/guidelines.xhtml

DISCUSSION

Main findings

We compared two cohorts of patients attending the ED following self-harm during a 3-month period in 2014 and 2015, following a £250,000 investment to extend LPS operating hours. Clear improvements were found in the proportion of patients receiving a psychosocial assessment as well as the time between ED attendance and psychosocial assessment, and reductions in self-discharge prior to assessment. There was a suggestion that the incidence of repeat self-harm declined, but we lacked statistical power to detect modest but clinically important effects. There was no evidence that the proportion of patients admitted to hospital decreased, however, the mean cost per patient (including repeat attendances) declined by approximately 14%. The findings from this analysis indicate that much of the additional £250,000 investment in liaison psychiatry services was offset by cost savings and improvements in management for self-harm patients. However, a larger study would be needed to confirm this.

The considerable decrease in referrals to the self-harm clinic and somewhat smaller increase in referrals to the Crisis team probably reflects improved service delivery for self-harm patients, as people who were previously discharged without an assessment were offered follow-up at the self-harm clinic the next day or within a couple of days; whereas with higher levels of assessment fewer people were referred to this clinic and more were referred to specialist mental health services.

Strengths and limitations

Our study has several strengths. First, rather than simply estimating the cost of the care of people who have self-harmed(16-18), we have sought to estimate the impact of additional investment in Liaison Psychiatry services and whether investment in this area results in cost savings e.g. arising from shorter periods of hospitalisation.

Second, our analysis was based on an unselected series of consecutive hospital presentations with self-harm and includes hospital admissions as well as ED attendances that did not lead to admission. Previous studies have been based on select patients groups e.g. those taking an overdose (8), or on small numbers of patients(19).

Lastly, we compared activity of the whole service, rather than attempting to identify patients who would have received the service prior to its inception, the approach used in the analysis of the Rapid Assessment Interface and Discharge (RAID) liaison psychiatry service in Birmingham, UK (11).

Nevertheless, there are a number of important limitations. Unlike the recent evaluation of the RAID service in Birmingham, UK, we did not measure the impact of other aspects of the Psychiatric Liaison team's activity; assessment of people who self-harm comprises only 40% of LPS referrals in the hospital in our study and a smaller proportion in the RAID evaluation. It is likely that the increase in LPS operating hours will provide a better service for all hospitalised patients with psychiatric morbidity.

For peer review only - http://bmjopen.bmj.com/site/about/guidelines.xhtml

In addition, we did not assess the impact of extended LPS on the entire package of care following presentation. In contrast, Sinclair (2011) estimated costs based on longer term follow-up(16). In addition to service costs associated with psychiatric or community mental health services during follow-up, there may also be measurable patient benefits when using a longer time horizon, but possibly also increased costs resulting from increased identification of (and referral for) psychiatric/social problems.

Furthermore, though our analysis was based on over 300 presentations before and after the introduction of extended liaison services, we had insufficient statistical power to demonstrate clinically important differences in some outcomes. For instance, the high variance between patients in length of stay and costs limits our ability to reach definitive conclusions about these outcomes. Also the observed 20% reduction in repeat self-harm episodes would be clinically important, but lacked statistical robustness.

Lastly, this analysis does not include a comparison hospital to control for secular trends in mental health and service provision. Despite the study limitations the major step-change in care provided a good opportunity to evaluate the impact of a more accessible LPS service, while detailed and relatively complete individual level patient data from a local self-harm register facilitated accurate estimation of repeat self-harm and secondary care costs for self-harm patients.

Findings in the context of the wider literature

Deficiencies and variations in the care of people presenting to hospital following self-harm have long been recognised (7, 8). Almost 20 years ago Kapur et al. (1998) explored differences in service delivery between hospitals with and without self-harm teams(7). In general, the level of service provision was considerably lower than in our study; Kapur reported that in hospitals without a self-harm team 39% of patients received a psychosocial assessment whereas in hospitals with such a team the proportion receiving an assessment was 46%. In our study 57% of patients were assessed in 2014, rising to 67% in 2015..

Due to different costing methods, cost implications are more difficult to compare across studies. Cost savings reported by Tadros et al evaluating the impact of the Birmingham RAID service, were in the range of £3.4 to £9.5 million a year(20). These estimates are based on a comparison of lengths of stays and rates of readmission only, and most of these savings come from reduced bed use among elderly patients. Our study only focused on LPS activities for self-harm patients, and potential benefits to and savings from care to elderly patients thus have not been included.

Sinclair et al examined economic findings based on a different approach, examining patterns of resource use and costs over a seven year follow-up period, and estimated how different factors contribute to total costs using regression analyses (16). Average costs were £2944 (SD £8438) per patient. Their study clearly illustrated the long term costs associated with the provision of health and social care to this patient group as social and mental health problems generally persist after episodes of crisis, and are largely managed by community health services. Appropriate and effective pathways of care thus generate additional

For peer review only - http://bmjopen.bmj.com/site/about/guidelines.xhtml

costs for self-harm patients, but also provide opportunity for more efficient i.e. cost saving solutions.

The only study that seems to allow direct comparison of cost-figures is by Kapur et al, reporting costs associated with hospital admission following deliberate self-poisoning, comparing hospitals with and without self-harm teams (18). Although they evaluated different service models, their estimates for hospital related costs were largely comparable (£510 vs. £390 for hospitals with and without self-harm teams, and £500 vs £391 for self-harm attendances before and after extension of service hours, respectively. They also demonstrated potential costs savings to be achieved by investing in more appropriate services for self-harm patients, although the particular interventions being compared were different.

Opportunities to improve health care and outcomes for self-harm patients also lie in identifying/developing effective psychiatric interventions for individual patients. For example, there is good evidence from systematic reviews that cognitive behavioural therapy based interventions reduce the incidence of repeat self-harm by almost one third(21). Patients benefiting from such interventions are likely to use less medical and community health services, and effective interventions thus have a high likelihood to be also cost-effective (22). As patients who frequently repeat self-harm generate the highest costs (16), psychiatric interventions reducing the risk of repeat self-harm may cost-saving on the longer term, as demonstrated for cognitive behaviour therapy(22).

Implications

Improving the quality of care in health services and investing in preventative services such as LPS is a difficult and continuous challenge, especially at a time of economic austerity. However, the Government's mental health strategy recognises the need for improved services at the interface between mental and physical health(23). Our evaluation emphasizes the importance of protecting and even expanding services where there is good evidence of improved clinical outcomes. It also highlights the need to adequately evaluate changes in health care models and associated costs before they are widely implemented (24). Increasing pressures on NHS budgets mean there is a danger of cutting funding for services that can potentially save very significant amounts of money for the local health economy in the long term. Apart from such financial constraints, the next question would be whether there are sufficient staff numbers available with the relevant skills and expertise to fill these roles.

There is limited evidence of the cost-effectiveness of liaison psychiatry services further work is needed in this area. In addition to health technology assessments evaluating (cost-)effectiveness of interventions for individual self-harm patients, high-quality cluster randomised trials are needed to reduce uncertainty regarding what are the most effective models of care for people experiencing mental health crisis.

For peer review only - http://bmjopen.bmj.com/site/about/guidelines.xhtml

ACKNOWLEDGEMENTS/FUNDING

The Bristol Self-harm Surveillance Register is funded by AWP NHS Trust and Bristol City Council. We thank all members of University Hospitals Bristol NHS Foundation Trust's Liaison Psychiatry team, in particular Salena Williams (team leader) and Kat Bramley and Peter Kennedy-Chapman (data collection) for their role in data collection, and Dr Robert Carroll and Dr Dee Knipe for data preparation.

The research was supported by the National Institute for Health Research (NIHR) Collaboration for Leadership in Applied Health Research and Care West (CLAHRC West) at University Hospitals Bristol NHS Foundation Trust. The views expressed are those of the author(s) and not necessarily those of the NHS, the NIHR or the Department of Health.

CONTRIBUTORSHIP

Dr Brent Opmeer contributed to the writing and critical reviewing of the manuscript, development of methods, analysis of data, interpretation of results, consensus meetings;

Dr Elsa Marques contributed to the design of the evaluation, acquisition and analysis of data, the writing and critical reviewing of the manuscript;

Ruta Margelyte contributed to the drafting, writing and critical reviewing of the manuscript;

Prof William Hollingworth and Prof David Gunnell conceived the project and contributed to the design of the evaluation, development of methods, analysis of data, interpretation of results, and writing and critical reviewing of the manuscript.

Each author listed on the manuscript has seen and approved the submission of this version of the manuscript and takes full responsibility for the manuscript.

COMPETING INTERESTS

We have read and understood BMJ Open policy on declaration of interests and declare that we have no competing interests.

DATA SHARING

Research data set is available on request for verification of the data analyses.

For peer review only

For peer review only - http://bmjopen.bmj.com/site/about/guidelines.xhtml

REFERENCES

1. Hawton K, Bergen H, Casey D, Simkin S, Palmer B, Cooper J, et al. Self-harm in England: a tale of three cities. Multicentre study of self-harm. Soc Psychiatry Psychiatr Epidemiol. 2007;42(7):513-21.
2. Cooper J, Steeg S, Bennewith O, Lowe M, Gunnell D, House A, et al. Are hospital services for self-harm getting better? An observational study examining management, service provision and temporal trends in England. BMJ Open. 2013;3(11):e003444.
3. Carroll R, Metcalfe C, Gunnell D. Hospital presenting self-harm and risk of fatal and non-fatal repetition: systematic review and meta-analysis. PLoS One. 2014;9(2):e89944.
4. Hawton K, Zahl D, Weatherall R. Suicide following deliberate self-harm: long-term follow-up of patients who presented to a general hospital. Br J Psychiatry. 2003;182:537-42.
5. Zahl DL, Hawton K. Repetition of deliberate self-harm and subsequent suicide risk: long-term follow-up study of 11,583 patients. Br J Psychiatry. 2004;185:70-5.
6. CG16 Self-harm: The short-term physical and psychological management and secondary prevention of self-harm in primary and secondary care: National Institute for Clinical Excellence (NICE); 2004 [Available from: https://www.nice.org.uk/guidance/cg16/evidence/full-guideline-189936541.
7. Kapur N, House A, Creed F, Feldman E, Friedman T, Guthrie E. Management of deliberate self poisoning in adults in four teaching hospitals: descriptive study. BMJ. 1998;316(7134):831-2.
8. Kapur N, House A, Dodgson K, May C, Creed F. Effect of general hospital management on repeat episodes of deliberate self poisoning: cohort study. BMJ. 2002;325(7369):866-7.
9. Carroll R, Metcalfe C, Steeg S, Davies NM, Cooper J, Kapur N, et al. Psychosocial Assessment of Self-Harm Patients and Risk of Repeat Presentation: An Instrumental Variable Analysis Using Time of Hospital Presentation. PLoS One. 2016;11(2):e0149713.
10. Bennewith O, Gunnell D, Peters T, Hawton K, House A. Variations in the hospital management of self harm in adults in England: observational study. BMJ. 2004;328(7448):1108-9.
11. Parsonage M, Fossey M. Economic evaluation of a liaison psychiatry service: Centre of Mental Health; 2011 [Available from: https://www.centreformentalhealth.org.uk/evaluation-liaison-psychiatry.
12. Craig P, Cooper C, Gunnell D, Haw S, Lawson K, Macintyre S, et al. Using natural experiments to evaluate population health interventions: guidance for producers and users of evidence: Medical Research Council (MRC); 2010 [Available from: https://www.mrc.ac.uk/documents/pdf/natural-experiments-guidance/.
13. Gilbody S, House A, Owens D. The early repetition of deliberate self harm. J R Coll Physicians Lond. 1997;31(2):171-2.
14. Fitzmaurice GM, Laird NM, Ware JH. Applied Longitudinal Analysis. Second Edition ed. Hoboken, New Jersey: John Wiley & Sons; 2011.

For peer review only - http://bmjopen.bmj.com/site/about/guidelines.xhtml

15. NHS reference costs 2014 to 2015: Department of Health (DH); 2015 [Available from: https://www.gov.uk/government/publications/nhs-reference-costs-2014-to-2015.
16. Sinclair JM, Gray A, Rivero-Arias O, Saunders KE, Hawton K. Healthcare and social services resource use and costs of self-harm patients. Soc Psychiatry Psychiatr Epidemiol. 2011;46(4):263-71.
17. Shepard DS, Gurewich D, Lwin AK, Reed GA, Jr., Silverman MM. Suicide and Suicidal Attempts in the United States: Costs and Policy Implications. Suicide Life Threat Behav. 2016;46(3):352-62.
18. Kapur N, House A, Dodgson K, May C, Marshall S, Tomenson B, et al. Management and costs of deliberate self-poisoning in the general hospital: A multi-centre study. Journal of Mental Health. 2002;11(2):223-30.
19. Sgobin SM, Traballi AL, Botega NJ, Coelho OR. Direct and indirect cost of attempted suicide in a general hospital: cost-of-illness study. Sao Paulo Med J. 2015;133(3):218-26.
20. Tadros G, Salama RA, Kingston P, Mustafa N, Johnson E, Pannell R, et al. Impact of an integrated rapid response psychiatric liaison team on quality improvement and cost savings: the Birmingham RAID model. The Psychaitrist. 2013;37:4-10.
21. Hawton K, Witt KG, Salisbury TL, Arensman E, Gunnell D, Hazell P, et al. Psychosocial interventions following self-harm in adults: a systematic review and meta-analysis. Lancet Psychiatry. 2016;3(8):740-50.
22. Byford S, Knapp M, Greenshields J, Ukoumunne O, Jones V, Thompson S, et al. Cost-effectiveness of brief cognitive behaviour therapy versus treatment as usual in recurrent deliberate self-harm: a decision-making approach. Psychological Medicine. 2003;33(6):977–86.
23. The mental health strategy for England - No Health Without Mental Health: a cross-government mental health outcomes strategy for people of all ages: Department of Health (DH); 2011 [Available from: https://www.gov.uk/government/uploads/system/uploads/attachment_data/file/213761/dh_124058.pdf.
24. Sinclair JM, Gray A, Hawton K. Systematic review of resource utilization in the hospital management of deliberate self-harm. Psychol Med. 2006;36(12):1681-93.

For peer review only - http://bmjopen.bmj.com/site/about/guidelines.xhtml

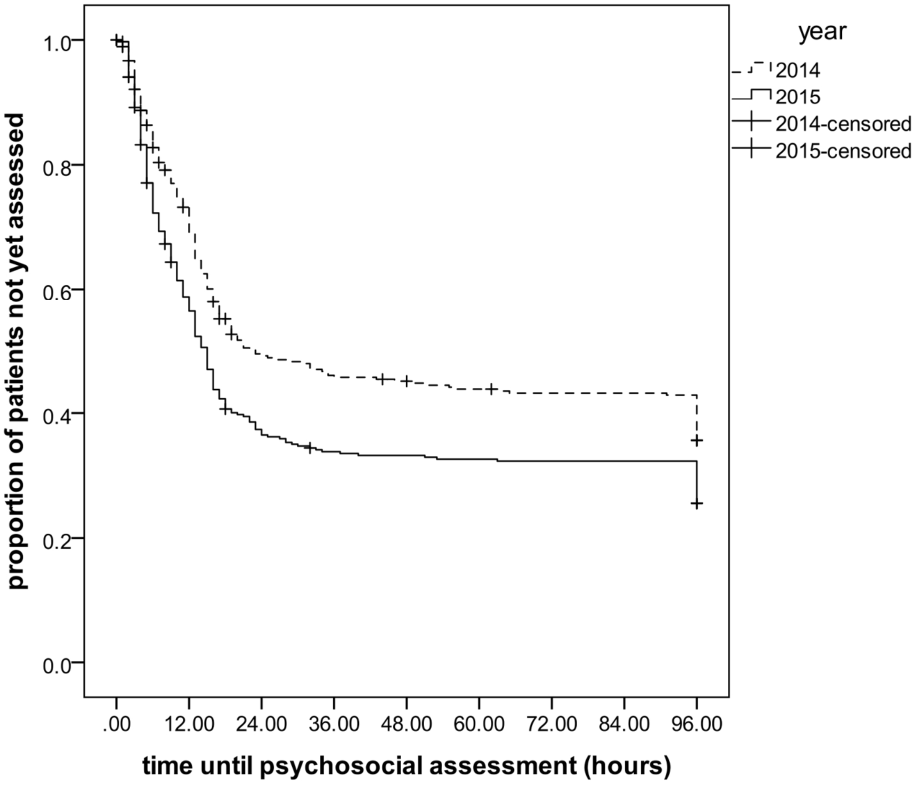

Figure 1 Time between arrival at ED and psychosocial assessment (log-rank test p-value: 0.001)

165x131mm (300 x 300 DPI)

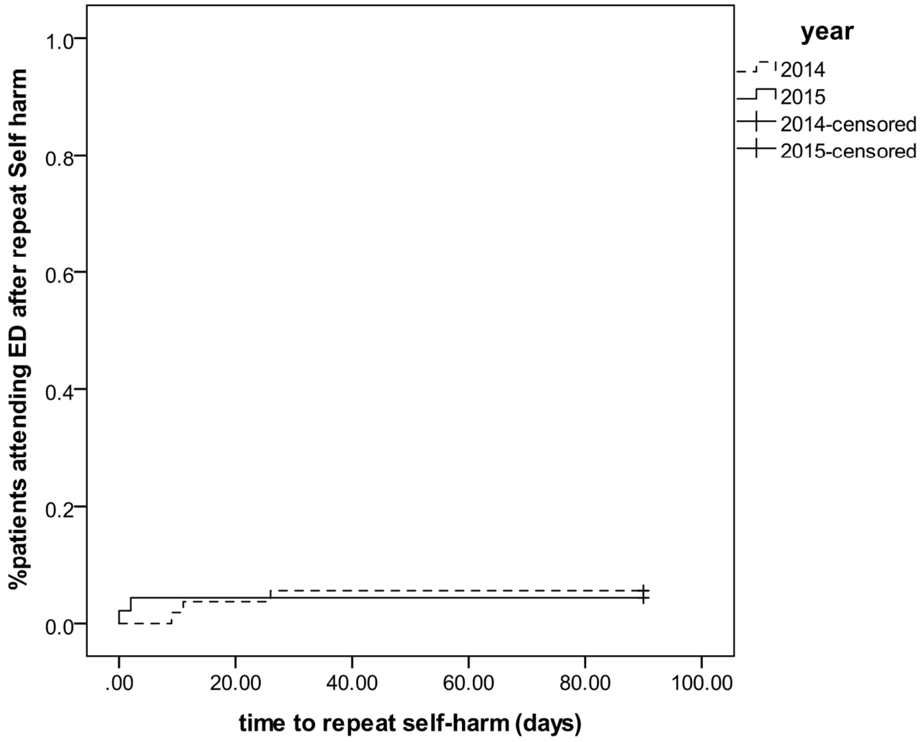

Figure 2 Cumulative incidence of repeat episodes of self-harm following the index attendance in patients with No previous self-harm (A);

165x131mm (300 x 300 DPI)

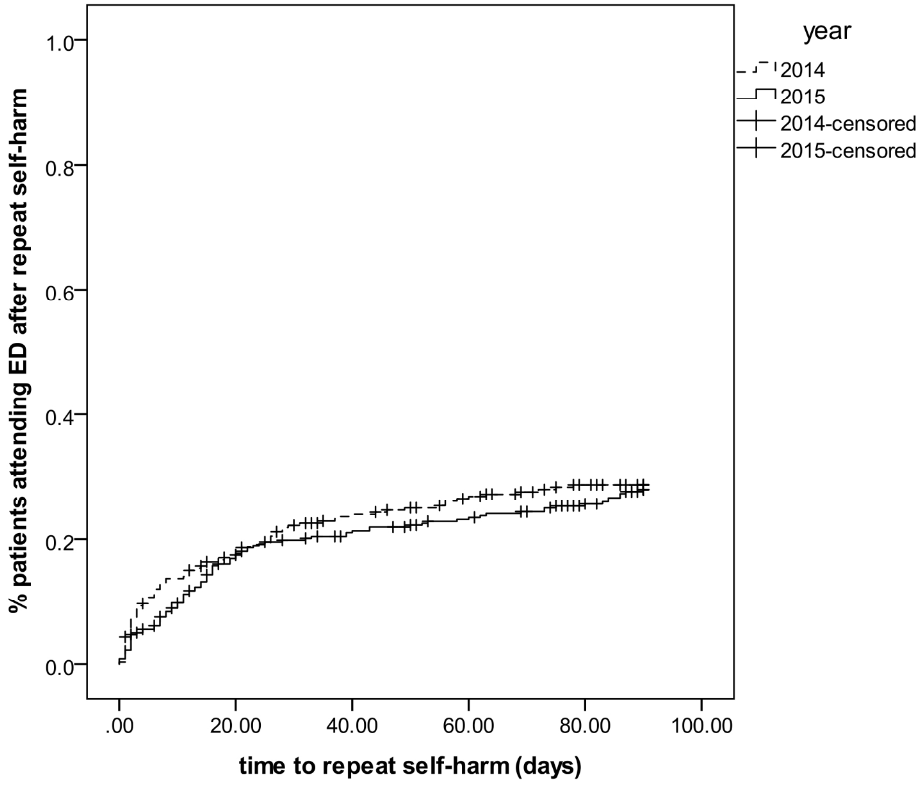

Previous self-harm (B);

165x131mm (300 x 300 DPI)

For peer review only

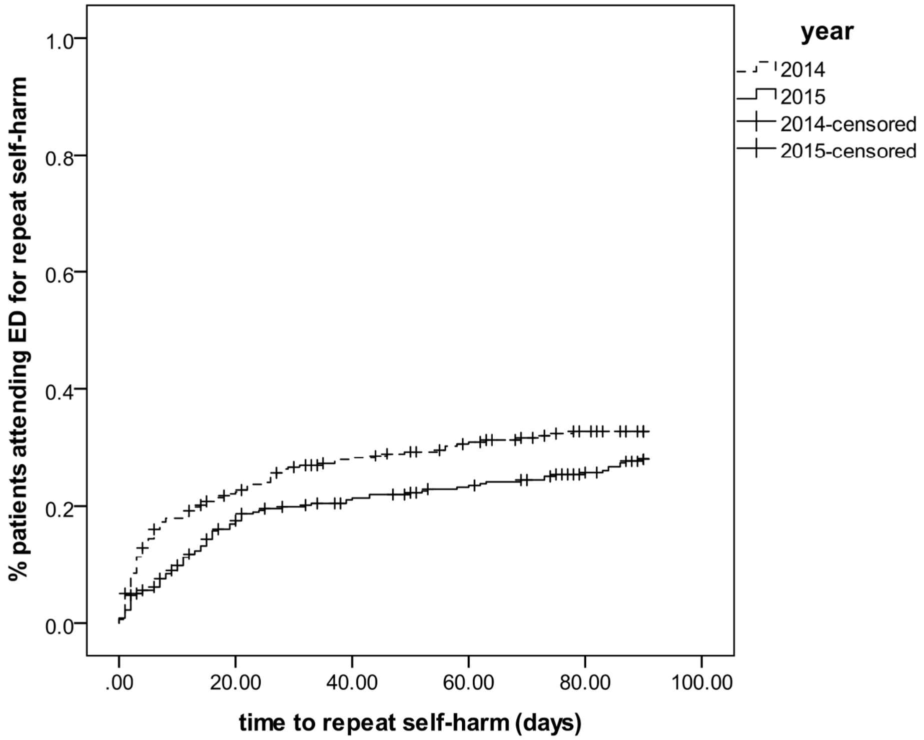

and All episodes of repeat self-harm < 90 days (C) for 2014 and 2015, based on Kaplan-Meier analyses.

165x131mm (300 x 300 DPI)

w only

STROBE Statement—checklist of items that should be included in reports of observational studies

	Item No	Recommendation
**Title and abstract**	1	(*a*) Indicate the study's design with a commonly used term in the title or the abstract **[See page 1 (title page) and method section of the abstract page 2]**
		(*b*) Provide in the abstract an informative and balanced summary of what was done and what was found **[See results section of abstract page 2]**
**Introduction**		
Background/rationale	2	Explain the scientific background and rationale for the investigation being reported **[See Introduction page 4]**
Objectives	3	State specific objectives, including any prespecified hypotheses **[See Introduction page 5]**
**Methods**		
Study design	4	Present key elements of study design early in the paper **[See Methods page 6]**
Setting	5	Describe the setting, locations, and relevant dates, including periods of recruitment, exposure, follow-up, and data collection **[See Methods pages 6]**
Participants	6	(*a*) *Cohort study*—Give the eligibility criteria, and the sources and methods of selection of participants. Describe methods of follow-up **[See Methods page 6]**<br>*Case-control study*—Give the eligibility criteria, and the sources and methods of case ascertainment and control selection. Give the rationale for the choice of cases and controls<br>*Cross-sectional study*—Give the eligibility criteria, and the sources and methods of selection of participants
		(*b*) *Cohort study*—For matched studies, give matching criteria and number of exposed and unexposed<br>*Case-control study*—For matched studies, give matching criteria and the number of controls per case
Variables	7	Clearly define all outcomes, exposures, predictors, potential confounders, and effect modifiers. Give diagnostic criteria, if applicable **[See Methods page 6 -7]**
Data sources/ measurement	8*	For each variable of interest, give sources of data and details of methods of assessment (measurement). Describe comparability of assessment methods if there is more than one group **[See Methods page 6 -7]**
Bias	9	Describe any efforts to address potential sources of bias **[See Methods page 6 (participants and data) and page 7 (statistical analysis)]**
Study size	10	Explain how the study size was arrived at **[See Methods page 6]**
Quantitative variables	11	Explain how quantitative variables were handled in the analyses. If applicable, describe which groupings were chosen and why **[See Methods page 7]**
Statistical methods	12	(*a*) Describe all statistical methods, including those used to control for confounding **[See Methods page 7]**
		(*b*) Describe any methods used to examine subgroups and interactions **[See Methods page 7]**
		(*c*) Explain how missing data were addressed **[See Methods page 6]**
		(*d*) *Cohort study*—If applicable, explain how loss to follow-up was addressed **[N/A]**<br>*Case-control study*—If applicable, explain how matching of cases and controls was addressed<br>*Cross-sectional study*—If applicable, describe analytical methods taking account of

For peer review only - http://bmjopen.bmj.com/site/about/guidelines.xhtml

		sampling strategy
		(*e*) Describe any sensitivity analyses **[See Methods page 8]**
**Results**		
Participants	13*	(a) Report numbers of individuals at each stage of study—eg numbers potentially eligible, examined for eligibility, confirmed eligible, included in the study, completing follow-up, and analysed **[See page 9;table 1]**
		(b) Give reasons for non-participation at each stage **[N/A]**
		(c) Consider use of a flow diagram **[Information presented in table 1]**
Descriptive data	14*	(a) Give characteristics of study participants (eg demographic, clinical, social) and information on exposures and potential confounders **[See page 9;table 1]**
		(b) Indicate number of participants with missing data for each variable of interest **[See table 1]**
		(c) *Cohort study*—Summarise follow-up time (eg, average and total amount) **[N/A]**
Outcome data	15*	*Cohort study*—Report numbers of outcome events or summary measures over time **[See pages 9-10;table 2;figures 1 and 2]**
		Case-control study—Report numbers in each exposure category, or summary measures of exposure
		Cross-sectional study—Report numbers of outcome events or summary measures
Main results	16	(*a*) Give unadjusted estimates and, if applicable, confounder-adjusted estimates and their precision (eg, 95% confidence interval). Make clear which confounders were adjusted for and why they were included **[See pages 9-10;table 2;figures 1 and 2]**
		(*b*) Report category boundaries when continuous variables were categorized **[N/A]**
		(*c*) If relevant, consider translating estimates of relative risk into absolute risk for a meaningful time period **[N/A]**
Other analyses	17	Report other analyses done—eg analyses of subgroups and interactions, and sensitivity analyses **[See pages 9-10;tables 3 and 4;figure 2]**
**Discussion**		
Key results	18	Summarise key results with reference to study objectives **[See page 11]**
Limitations	19	Discuss limitations of the study, taking into account sources of potential bias or imprecision. Discuss both direction and magnitude of any potential bias **[See page 11]**
Interpretation	20	Give a cautious overall interpretation of results considering objectives, limitations, multiplicity of analyses, results from similar studies, and other relevant evidence **[See pages 11-12]**
Generalisability	21	Discuss the generalisability (external validity) of the study results **[See page 11]**
**Other information**		
Funding	22	Give the source of funding and the role of the funders for the present study and, if applicable, for the original study on which the present article is based **[See pages 13]**

*Give information separately for cases and controls in case-control studies and, if applicable, for exposed and unexposed groups in cohort and cross-sectional studies.

Note: An Explanation and Elaboration article discusses each checklist item and gives methodological background and published examples of transparent reporting. The STROBE checklist is best used in conjunction with this article (freely available on the Web sites of PLoS Medicine at http://www.plosmedicine.org/, Annals of Internal Medicine at http://www.annals.org/, and Epidemiology at http://www.epidem.com/). Information on the STROBE Initiative is available at www.strobe-statement.org.

For peer review only
