# Supplementary material for: Extending the liaison psychiatry service in a large hospital in the UK: a before and after evaluation of the economic impact and patient care following ED attendances for self-harm
Source: BMJ Open. 2017 Aug 21;7(8):e016906. doi: 10.1136/bmjopen-2017-016906 (PMC5724115; doi:10.1136/bmjopen-2017-016906)
Supplement: Reviewer comments [file bmjopen-2017-016906.reviewer_comments.pdf]

PEER REVIEW HISTORY

BMJ Open publishes all reviews undertaken for accepted manuscripts. Reviewers are asked to complete a checklist review form (**http://bmjopen.bmj.com/site/about/resources/checklist.pdf**) and are provided with free text boxes to elaborate on their assessment. These free text comments are reproduced below.

ARTICLE DETAILS

**TITLE (PROVISIONAL)**	Extending the liaison psychiatry service in a large hospital in the UK: a before and after evaluation of the economic impact and patient care following Emergency Department attendances for self-harm
**AUTHORS**	Opmeer, Brent; Hollingworth, William; Marques, Elsa; Margelyte, Ruta; Gunnell, David

VERSION 1 - REVIEW

**REVIEWER**	Jens Klotsche<br>German Rheumatism Research Center Berlin, Germany
**REVIEW RETURNED**	17-Apr-2017

**GENERAL COMMENTS**	Patients<br>- Please report more details about patient recruitment. The Strobe guideline may guide the report.<br><br>Data quality/ missing data.<br><br>Some more details about missing data should be reported.<br>- What is the proportion of missing data? (average, range,…)<br>- What problems were evident resolved? How did you impute missing data?<br><br>Statistical analyses<br>- The median is used for characterization of the distribution of continuously distributed variables. What is the rationale? When did you use the median rather than the mean value?<br>- Did you report or analyze the differences in median or the median of the difference in a variable that is longitudinal measured (see evaluation of impact, first three lines)?<br>- Was the proportional hazard assumption tested for the Cox-proportional hazard models?<br>- How did you model repeated self-harm attendances? Frailty models may be used for the analyses of repeated events.<br>- Bootstrap confidence intervals were used for cost data. Please report the number of bootstrap replications. What method was used for estimating the bootstrap confidence intervals?<br>- Sensitivity analyses for cost data were conducted. It is totally vague how the authors performed the analyses? Was the sensitivity analyses based on a Monte-Carlo simulation assuming a probability distribution for the considered parameters? What does varying cost parameters mean?<br>- Figure 2: Figure 2 shows the cumulative incidence estimated by the Kaplan-Meier method.

**REVIEWER**	Harriet Bickley<br>University of Manchester, England, UK<br><br>I have not collaborated with any of the authors. However, I do collaborate with researchers who the authors have also collaborated with, but on separate projects.<br><br>I have also undertaken approximately 5 days paid administrative support work (specifically minute-taking and minute-writing up) on a different study to the one I am reviewing, but which one of the authors is a Principal Investigator on.
**REVIEW RETURNED**	18-Apr-2017

**GENERAL COMMENTS**	Review for BMJ Open ‘Extending the liaison psychiatry service: a before and after evaluation of the economic impact in Emergency Department attendances for self-harm’, Opmeer et al, April 2017<br><br>Description: This paper describes an evaluation of the financial and clinical impact of extending the hours of a liaison psychiatry service (LPS) serving an Emergency Department in South West England. They assess a comprehensive range of outcome measures covering many clinically important and measurable outcomes. The study benefits from a large sample size. This is a very timely and useful study for hospitals and healthcare commissioners across the NHS, providing evidence on whether increased liaison psychiatry spending can result in cost savings elsewhere, whilst improving patient care. There appears to be only one other published UK study that has reported on this scenario (Parsonage et al, 2011).<br><br>The authors assess 5 process measures: proportion of patients receiving a psychosocial assessment (this is important because Kapur et al, 2013, PLoS ONE found it might reduce self-harm repetition by 40%; also NICE Clinical Guidelines in 2004 & Royal College of Psychiatry guidelines say 100% of people attending ED with self-harm should receive a detailed psychosocial assessment); waiting times from attendance to assessment; proportion of patients self-discharging from the ED without assessment; proportion of episodes admitted to a ward; average length of hospital stay.<br><br>They assess 3 patient outcome measures: proportion of patients with repeat self-harm ED attendances; number of repeat self-harm ED attendances; time to repeated self-harm attendance.<br><br>Two average cost measures were also calculated: mean cost per self-harm ED attendance; mean cost per patient associated with the index episode.<br><br>The liaison psychiatry services more than doubled their weekly operational hours, from 40 to 98 hours, expanding a 5 day service to a 7 day service. Since LPS are the service providing most or all of the psychosocial assessments, this increased availability could be expected to increase the proportion of ED self-harm presentations receiving such an assessment. There may also be a reduction in the average waiting time for an assessment, which has the dual benefits of a potentially more rapid reduction in a patient’s distress, and reducing pressure on other aspects of the service, eg availability of medical beds.<br><br>The good news stories for patients out of this research are that

	patients were more likely to receive a psychosocial assessment, and were more likely to receive it sooner after presenting to the ED unit, and the patient self-discharge rate was lower. There was also a reduction in repetition of ED presentation of self-harm. This study therefore provides good evidence of improved clinical outcomes.<br><br>Abstract: The authors accurately describe strengths and limitations of the study.<br>Introduction: The authors clearly explain why self-harm research is important, and what this paper adds to the evidence base.<br><br>Method: The statistical methods and variables are clearly outlined.<br><br>Results: The authors describe differences in the patient groups in the before and after samples. The results state that there was a large decrease in referrals to the self-harm clinic and an increase in referrals to other community mental health teams.<br><br>Question1: In the Method outcomes section the authors mention use of a locally developed 'matrix risk assessment tool' (p6, line 47), but it is not clear if this is a validated tool. Could this be clarified in the method section? It would be useful to know whether this is a simplistic risk assessment tool or whether it is a more comprehensive psychosocial assessment.<br><br>Question 2: Could the authors explain what the self-harm clinic is (p9 line56)?<br><br>Question 3: Could the authors provide an explanation in the discussion section of why there was a large decrease in referrals to the self-harm clinic and corresponding increase in referrals to the intensive mental health team? Was this due to the new LPS staff?<br><br>Question 4: In Figure 1, is the unit of time until psychosocial assessment supposed to be measured in days? I am finding it difficult to relate Figure 1 (p22 line 31) to the numbers in the text where the median time in is measured in hours and minutes (p9, line 41).<br><br>Question 5: The text (p9 line 38) states that the proportion of patients receiving a psychosocial assessment outside the core LPS hours was 28%. I am assuming that those assessments were carried out by ED staff rather than LPS staff, but could the authors clarify this?<br><br>Question 6: What does 'BRI' in Table 3 stand for; is it Bristol Royal Infirmary?<br><br>Question 7: What does 'nr' in Table 4 stand for?<br><br>Question 8: Staffing implications: To extend the LPS service from 40 to 98 hours, Bristol employed 4 additional full-time psychiatric liaison nurses across 3 months. Would these nurses have been hired through an agency? If so, I'm assuming the cost of employing them would have been much higher per hour than if they had been employed by the local NHS Trust on a longer-term contract, and would have implications for the costing model.<br><br>Question 9: If LPS were to expand across England and Wales, are there enough staff to fill the roles?

	Question 10: Am I correct in assuming that the LPS service was scaled back to 40 hours per week from April 2015? If so, it may be interesting for Jan-Jun 2016 data to be compared to the 2014 and 2015 data, ie once the intervention was removed again.<br><br>Question 11: In the introduction the authors refer to a similar study which took place in Birmingham (Parsonage et al, 2011)? Can the current study, or a future Bristol study, produce the same statistic as the Birmingham study, ie reduced bed use with overall benefit to cost ratio of more than 4:1?<br><br>Question 12: Where did the extra £250,000 from the CCG to enable the extended LPS service come from, and was there corresponding loss from elsewhere in the mental health budget?<br><br>Comment 1: Whilst it is commendable to reduce the delay between ED attendance with self-harm and a psychosocial assessment, if a patient attends ED intoxicated from drugs or alcohol, or in the midst of psychosis, then there may be a clinically necessary delay between ED presentation and commencement of a psychosocial assessment.<br><br>Comment 2: It is a shame that the £144,600 hospital costs estimated to be saved is not more than the £250,000 additional investment in the LPS. However, the savings may outweigh the investment if the impact on primary and community care services were to be included in future work. The authors say that such service could 'potentially save very significant amounts of money for the local health economy in the long-term' but I worry that this may be overstating the findings of the current study.<br><br>Comment 3: Would an ideal liaison psychiatry service provide be a 24-hour service? Are there any non-financial arguments against an LDS becoming a 24-hour service? It would be interesting to know the ED staff's opinions on the benefits of having increased access to the LPS service.<br><br>Comment 4: It would be useful to know what hours LPS are available across the country to see the potential for extending LPS hours of service across the NHS.<br><br>Minor typographic errors:<br>Abstract: the first sentence should include 'services seem' or 'service seems' (page 2, line 41).<br>Abstract: self-harm is misspelt as slef-harm (p2, line 42).<br>Methods: there should be a hyphen between Kaplan and Meier (p7, line 44).<br>Results: add in a missing % sign after '68' (p9 line 35/6).<br>Results: the proportion of psychosocial assessments taking place outside of 2014 LPS working hours in 2014 is 28% in the text (p9, line 38) but 29% in Table 2 (p15, line 16). It looks like 29% is the correct number.

VERSION 1 – AUTHOR RESPONSE

Comments Reviewer: 1

Patients
- Please report more details about patient recruitment. The Strobe guideline may guide the report.
As this is a register-based study, there was no actual patient recruitment. Key information for all hospital presentations for self-harm (sociodemographic characteristics and clinical details) were documented on the pro forma used to collect data for the Self-harm Register, and subsequently entered in the self-harm surveillance register's (SHSR) database.

Data quality/ missing data.

Some more details about missing data should be reported.
- What is the proportion of missing data? (average, range,…)
Overall, details in patient characteristics and patient care were accurately documented in the self-harm register. The number (%) of people with missing data are reported in Tables 1 and 2. For key variables, e.g. whether an assessment was performed, previous self-harm, time of assessment, time of discharge, outcome of ED attendance, completeness ranged from 0% (assessment performed/ outcome of attendance) to 9.7% (previous self-harm).
We reported these findings re missing data as part of the results section (page 10).
To avoid bias due to observations excluded from statistical analyses, we used multiple imputation (SAS Proc MI and Proc MIanalyze) to impute missing data and estimated a pooled effect across imputed datsets. We explained this in the methods (page 7):
"Multiple imputation (SAS Proc MI and Proc MIAnalyze) was used, with 15 imputation rounds, to avoid exclusion of observations due to missing data in multivariable analyses (see below)".

- What problems were evident resolved? How did you impute missing data?
Audits reveal SHSR case ascertainment is >95%. Data extracted from the SHSR were checked for inconsistencies, and where evident these were resolved.
The two main types of inconsistency that were identified were date errors (e.g. dates of discharge preceded data of attendance) and variables reflecting composite questions, where the first variable was missing (e.g. admitted to ward), but the second variable was completed (e.g. date of admission to ward). In these evident cases, inconsistent or missing values were corrected with consistent values.
We have elaborated this section accordingly (page 6).

Statistical analyses
- The median is used for characterization of the distribution of continuously distributed variables. What is the rationale? When did you use the median rather than the mean value?
We calculated and reported medians for the variables reflecting the duration between two occurrences, e.g. between ED attendance and medical assessment. First the distribution of these variables is generally quite skewed, and medians better reflect the middle of the distribution. Second, by reporting medians, results are more consistent with how outcomes are reported in time to event analyses (Kaplan Meier), i.e. median time to event. Although there will be some differences when reporting means, this would not alter the interpretation of our results.

- Did you report or analyze the differences in median or the median of the difference in a variable that is longitudinal measured (see evaluation of impact, first three lines)?
We reported the differences between the medians of the outcome variable.

- Was the proportional hazard assumption tested for the Cox-proportional hazard models?
Yes, we inspected LML plots and judged the curves as parallel.

- How did you model repeated self-harm attendances? Frailty models may be used for the analyses of repeated events.
We used the SAS PHREG procedure to model time to event outcomes, allowing for multiple events per observation, and using Sandwich estimator to adjust for correlated observations within the same patient, as reported in the statistics section. We now explicitly mention the SAS PHREG procedure used for this analysis (page 8).

- Bootstrap confidence intervals were used for cost data. Please report the number of bootstrap replications. What method was used for estimating the bootstrap confidence intervals?
We used 1000 bootstrap replications using the simple bootstrap procedure and reported 95% BCI in SPSS. We added this information in the text (page 8)

- Sensitivity analyses for cost data were conducted. It is totally vague how the authors performed the analyses? Was the sensitivity analyses based on a Monte-Carlo simulation assuming a probability distribution for the considered parameters? What does varying cost parameters mean?
Univariate sensitivity analyses explored the robustness of the results by varying key parameters or assumptions one by one in the cost analysis. In four of these sensitivity analyses, we used different (25% lower and 25% higher) unit costs for the LPS referral (table 4 id 3 and 4); and hospital ward day (table 4 id 5 and 6). We conducted sensitivity analysis, using a general ward day cost for all hospital days (table 4 id 2), and assuming a different unit costs for LPS referrals where a patient is assessed by a liaison nurse (-25%) or a psychiatrist (+25%) (table 4 id 1). We clarified this in this section on page 8.

- Figure 2: Figure 2 shows the cumulative incidence estimated by the Kaplan-Meier method.
Indeed, figures A-C show the cumulative incidence estimated by the KM analysis, and we adjusted the caption accordingly.

Reviewer: 2

Question1: In the Method outcomes section the authors mention use of a locally developed 'matrix risk assessment tool' (p6, line 47), but it is not clear if this is a validated tool. Could this be clarified in the method section? It would be useful to know whether this is a simplistic risk assessment tool or whether it is a more comprehensive psychosocial assessment.
This is not a validated tool. It is a crude risk assessment tool for completion by (non psychiatry) Emergency Department medical and nursing staff at triage. Patients are categorised as green (low) , amber (intermediate) or red (high) risk on the matrix; these levels guide clinical staff in deciding whether a patient should be referred for an immediate psychosocial assessment. We added an explanation to clarify this (page 6)

Question 2: Could the authors explain what the self-harm clinic is (p9 line56)?
This is an outpatient clinic for low-risk patients who are discharged from the ED without an immediate assessment; they are invited to return several days later for review by a member of the LPS.

Question 3: Could the authors provide an explanation in the discussion section of why there was a large decrease in referrals to the self-harm clinic and corresponding increase in referrals to the intensive mental health team? Was this due to the new LPS staff?
We added the following section to explain this shift in referrals following the attendance to the emergency department in the discussion section:
"The considerable decrease in referrals to the self-harm clinic and somewhat smaller increase in referrals to the Crisis team probably reflects improved service delivery for self-harm patients, as

people who were previously discharged without an assessment were offered follow-up at the self-harm clinic the next day or within a couple of days; whereas with higher levels of assessment fewer people were referred to this clinic and more were referred to specialist mental health services. (page 12)

Please note the Crisis team was re-named the Intensive mental health team during the study period, we have therefore combined data on referral to this team into a single row in Table 2

Question 4: In Figure 1, is the unit of time until psychosocial assessment supposed to be measured in days? I am finding it difficult to relate Figure 1 (p22 line 31) to the numbers in the text where the median time in is measured in hours and minutes (p9, line 41).
The author correctly noted that the unit expressing time to psychosocial assessment should be hours, instead of days. We adjusted the label as well as the tics on this axis to reflect this.

Question 5: The text (p9 line 38) states that the proportion of patients receiving a psychosocial assessment outside the core LPS hours was 28%. I am assuming that those assessments were carried out by ED staff rather than LPS staff, but could the authors clarify this?
These were performed by mental health staff such as on-call psychiatrists and the local crisis service. We do not categorise assessments undertaken by ED staff as psychosocial assessments.

Question 6: What does ‘BRI’ in Table 3 stand for; is it Bristol Royal Infirmary?
We have replaced “BRI” with “Hospital”.

Question 7: What does ‘nr’ in Table 4 stand for?
Nr was a numbering to identify and refer to each analysis. We now changed this heading to (Analysis) id to avoid this confusion.

Question 8: Staffing implications: To extend the LPS service from 40 to 98 hours, Bristol employed 4 additional full-time psychiatric liaison nurses across 3 months. Would these nurses have been hired through an agency? If so, I’m assuming the cost of employing them would have been much higher per hour than if they had been employed by the local NHS Trust on a longer-term contract, and would have implications for the costing model.
No, they were employed as new staff members within the LPS.

Question 9: If LPS were to expand across England and Wales, are there enough staff to fill the roles?
This is a good point, although related to rather practical implications and potential obstacles when this evidence warrants the conclusion that LPS expansion should be considered for all similar services across the UK and Wales. We added the following sentence to address this in the conclusion. ”Apart from such financial restraints, the next question would be whether there are sufficient staff numbers available with the relevant skills and expertise to fill these roles.” (page 12).

Question 10: Am I correct in assuming that the LPS service was scaled back to 40 hours per week from April 2015? If so, it may be interesting for Jan-Jun 2016 data to be compared to the 2014 and 2015 data, ie once the intervention was removed again.
No, the extension continued after the 3-month inception period for the cohort; actually, the results of this evaluation contributed to the decision to further scale up the service hours to 24/7 (due to commence in 2018)

Question 11: In the introduction the authors refer to a similar study which took place in Birmingham (Parsonage et al, 2011)? Can the current study, or a future Bristol study, produce the same statistic as the Birmingham study, ie reduced bed use with overall benefit to cost ratio of more than 4:1?
Unfortunately the data available from the self-harm register provide limited detail to systematically

analyse and compare bed use by self-harm patients. The estimated figures for length of hospital admissions, do not suggest a reduction of length of stay of a similar magnitude. As for the comparison with the study by Parsonage (2011)evaluating the RAID psychiatric liaison service in Birmingham: their study evaluated the full range of service delivery to all patients referred to the liaison psychiatric service, and thus hardly comparable to our study. For instance, most of the cost savings reported by Parsonage come from reduced bed use among elderly patients – a very different group of patients as compared to self-harm patients. In a fuller evaluation of the Bristol LPS investment, it will be important to document and analyse bed use and other cost drivers in more detail to allow estimation and comparison of this benefit-to-cost ratio.

Question 12: Where did the extra £250,000 from the CCG to enable the extended LPS service come from, and was there corresponding loss from elsewhere in the mental health budget?
WH: The £250,000 came from CCG funds. It is not clear whether this resulted in cuts elsewhere (but given NHS funding constraints that's almost inevitable).
DG: Our understanding is that this was new funding and did not involve cuts in other aspects of the mental health budget (indeed the service is commissioned from the local cute trust, not the mental health trust).

Comment 1: Whilst it is commendable to reduce the delay between ED attendance with self-harm and a psychosocial assessment, if a patient attends ED intoxicated from drugs or alcohol, or in the midst of psychosis, then there may be a clinically necessary delay between ED presentation and commencement of a psychosocial assessment.
We agree that there may be good clinical reasons to delay the psychosocial assessment of some patients to allow them to recover from acute intoxication and the clinical consequences of their self-harm, so it is unrealistic to expect 100% psychosocial assessments to take place immediately. When evaluating service delivery for SH patients by ED/LPS, one could take this into account by identifying and correcting for such patients. However, our data contained insufficient medical details to facilitate such a sensitivity analysis.

Comment 2: It is a shame that the £144,600 hospital costs estimated to be saved is not more than the £250,000 additional investment in the LPS. However, the savings may outweigh the investment if the impact on primary and community care services were to be included in future work. The authors say that such service could ‘potentially save very significant amounts of money for the local health economy in the long-term’ but I worry that this may be overstating the findings of the current study.
We agree that this statement seems to link our findings directly to “potential very large savings in the local health economy”, which is beyond the evidence from this study; we therefore adjusted this statement to emphasise the importance of systematic evaluation prior to implementing organisational changes in health care services. ”It also highlights the need to adequately evaluate changes in health care models and associated costs before they are widely implemented. Despite the increasing pressure on NHS budget, without such evidence there is a danger of cutting funding for services that can potentially save very significant amounts of money for the local health economy in the long term” (page 12)

Comment 3: Would an ideal liaison psychiatry service provide be a 24-hour service? Are there any non-financial arguments against an LDS becoming a 24-hour service? It would be interesting to know the ED staff’s opinions on the benefits of having increased access to the LPS service.
This is a good question and requires further study. As service hours are further extended (any) marginal benefits are likely to decline. The further challenge with further extending operating hours are the difficulties with staff recruitment mentioned above, plus the additional management costs for senior staff in team management roles.

Comment 4: It would be useful to know what hours LPS are available across the country to see the

potential for extending LPS hours of service across the NHS.
This is also a good point. Service availability is very varied across the country. Some services provide 24 hour care and in other parts of the country there is no psychiatric liaison team at all.

VERSION 2 – REVIEW

**REVIEWER**	Jens Klotsche<br>German Rheumatism Research Center Berlin, Germany
**REVIEW RETURNED**	24-Jun-2017

**GENERAL COMMENTS**	The authors adequately addressed all my concerns. I do not have any further comment.

**REVIEWER**	Harriet Bickley<br>University of Manchester, UK.<br><br>I received payment for attending and typing up minutes from two days of meetings for a different study, but which has a Principal Investigator in common with an author on this paper. I have also spent one day looking up information for a colleague who works on a different project with one of the authors. I have not otherwise collaborated with any of the authors, have not published with any of the authors and am not from the same institution as any of the authors.
**REVIEW RETURNED**	23-Jun-2017

**GENERAL COMMENTS**	The authors have appropriately addressed the comments and questions which I gave after reading their first draft.
